# Increased Expression of Interleukin-1 Receptor Characterizes Anti-estrogen-Resistant ALDH^+^ Breast Cancer Stem Cells

**DOI:** 10.1016/j.stemcr.2020.06.020

**Published:** 2020-07-23

**Authors:** Aida Sarmiento-Castro, Eva Caamaño-Gutiérrez, Andrew H. Sims, Nathan J. Hull, Mark I. James, Angélica Santiago-Gómez, Rachel Eyre, Christopher Clark, Martha E. Brown, Michael D. Brooks, Max S. Wicha, Sacha J. Howell, Robert B. Clarke, Bruno M. Simões

**Affiliations:** 1 Manchester Breast Centre, Division of Cancer Sciences, University of Manchester, Manchester M20 4GJ, UK; 2 Technology Directorate, Institute of Systems, Molecular & Integrative Biology, University of Liverpool, Liverpool L69 7ZB, UK; 3 Applied Bioinformatics of Cancer Group, University of Edinburgh Cancer Research UK Centre, Edinburgh EH4 2XR, UK; 4 Comprehensive Cancer Center, University of Michigan, Ann Arbor, MI 48109, USA

**Keywords:** breast cancer stem cells, anti-estrogens, ALDH^+^ cells, IL1R1, dormancy

## Abstract

Estrogen-receptor-positive breast tumors are treated with anti-estrogen (AE) therapies but frequently develop resistance. Cancer stem cells (CSCs) with high aldehyde dehydrogenase activity (ALDH^+^ cells) are enriched following AE treatment. Here, we show that the interleukin-1β (IL-1β) signaling pathway is activated in ALDH^+^ cells, and data from single cells reveals that AE treatment selects for IL-1 receptor (IL1R1)-expressing ALDH^+^ cells. Importantly, CSC activity is reduced by an IL1R1 inhibitor in AE-resistant models. Moreover, IL1R1 expression is increased in the tumors of patients treated with AE therapy and predicts treatment failure. Single-cell gene expression analysis revealed that at least two subpopulations exist within the ALDH^+^ population, one proliferative and one quiescent. Following AE therapy the quiescent population is expanded, which suggests CSC dormancy as an adaptive strategy that facilitates treatment resistance. Targeting of ALDH^+^IL1R1^+^ cells merits testing as a strategy to combat AE resistance in patients with residual disease.

## Highlights

• Anti-estrogen-resistant ALDH^+^ cells have increased CSC activity in ER^+^ tumors
• The IL1R1-expressing ALDH^+^ CSC population expands after anti-estrogen treatment
• IL1R1 expression predicts anti-estrogen treatment failure
• Targeting of ALDH^+^IL1R1^+^ cells reverses anti-estrogen resistance

## Teaser

In this study, Simões, Clarke and colleagues show that anti-estrogen treatments select for ALDH^+^ breast cancer stem cells expressing *IL1R1*, which can be targeted by a specific inhibitor. Increased expression of *IL1R1* is observed in the tumors of patients treated with anti-estrogen therapy, suggesting that targeting of ALDH^+^IL1R1^+^ cells will help combat therapy resistance.

## Introduction

Breast cancer (BC) represents 25% of all cancer diagnoses and is the fifth most common cause of death in women worldwide. Approximately 80% of BCs are positive for estrogen receptor expression (ER^+^ tumors) and are treated with anti-estrogen (AE) adjuvant therapies such as tamoxifen or fulvestrant. Despite the clear benefit of these drugs at reducing tumor recurrence, *de novo* or acquired resistance often occurs (Pan et al., 2017).

Cancer stem cells (CSCs) are a cellular population endowed with self-renewal properties, which are responsible for tumor progression and metastasis (Reya et al., 2001). Aldehyde dehydrogenase (ALDH) activity is reported to be a CSC marker in human BC cells (Ginestier et al., 2007). ALDH^+^ cells are ER-negative and likely to be resistant to the direct effects of AE therapy (Honeth et al., 2014). We have previously established that ALDH^+^ cells drive therapeutic resistance in ER^+^ BC tumors (Simões et al., 2015).

Intra-tumor heterogeneity within BCs hinders accurate diagnosis and effective treatment. Understanding of the cellular diversity within the CSC population, especially at the single-cell level, is limited. Given the importance of ALDH^+^ cells in promoting AE resistance, we investigated the gene expression pattern of this cellular population at the single-cell level. This study reveals a previously uncharacterized level of heterogeneity within AE-resistant CSCs and identifies IL1R1 as a potential target in refractory and dormant BCs.

## Results

### ALDH^+^ Cells from AE-Treated ER^+^ BCs Have Greater Breast CSC Activity Than ALDH^−^ Cells

**Figure 1:**
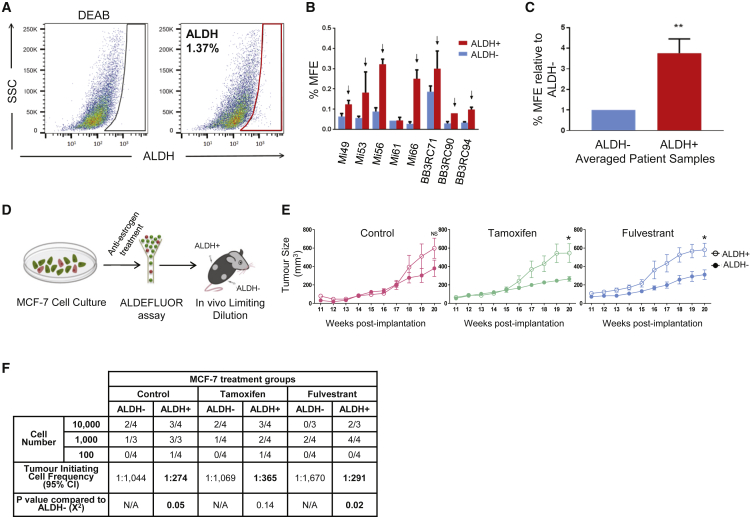
AE-Treated ALDH^+^ Cells from ER^+^ BC Cells Have Greater BCSC Activity Than ALDH^−^ Cells *In Vitro* and *In Vivo* (A) Representative fluorescence activated cell sorting (FACS) plot showing the ALDH^+^ population identified through the Aldefluor assay for an individual patient sample. ALDH^+^ cells (red gate) were discriminated from ALDH^−^ cells using the diethylaminobenzaldehyde (DEAB) control. (B) Bar chart shows mammosphere-forming efficiency (MFE) percentage of ALDH^+^ cells (red) and ALDH^−^ cells (blue) from ER^+^ metastatic BCs undergoing AE therapies. (C) Bar chart illustrates fold change in MFE percentage between ALDH^+^ and ALDH^−^ cells across eight different patient samples. (D) Schematic overview of the *in vivo* transplantation assay to test tumor formation capacity between ALDH^+^ and ALDH^−^ MCF-7 cells. MCF-7 cells were pre-treated *in vitro* for 6 days with control (ethanol), tamoxifen (1 μM) or fulvestrant (0.1 μM) followed by the Aldefluor assay. ALDH^+^ and ALDH^−^ cells were FACS sorted, counted using trypan blue, and engrafted into the left and right flank, respectively, of the same NSG mice. (E) Averaged tumor growth from control (pink; left panel), tamoxifen (green; middle panel), or fulvestrant-treated (blue; right panel) cells. 1,000 ALDH^+^ (hollow circles) and 1,000 ALDH^−^ (filled circles) cells are represented. ^∗^p ≤ 0.05 (two-tail, two-sample equal-variance t test). Number of mice per condition = 4 (vehicle-treated mice, n = 3). Data shown as mean ± SEM. (F) Table shows extreme limiting dilution analysis from *in vivo* injections of ALDH^+^ and ALDH^−^ cells (10,000; 1,000; 100 cells) to assess tumor-initiating cell frequency. Tumor growth was assessed at week 20 and is represented as mice positive for growth/mice tested for each cell number. See also Figure S1.

Previous research reported by our group (Simões et al., 2015) established that AE treatment of BC patient-derived xenograft tumors in mice enriches for breast CSCs (BCSCs) with high ALDH enzymatic activity. To further investigate this AE-resistant population, we isolated ALDH^+^ and ALDH^−^ cells from eight metastatic ER^+^ BCs undergoing AE therapies. There was significant inter-individual variation in the percentage of ALDH^+^ cells (range 0.32%–27.3%) (Figures 1A and S1A). Importantly, ALDH^+^ cells exhibited significantly greater BCSC activity as assessed by mammosphere formation than ALDH^−^ cells in seven out of eight patient samples, and in four of these samples the mammosphere-forming efficiency (MFE) was increased by more than 3-fold (Figure 1B). On average, ALDH^+^ cells from the eight metastatic BC samples showed 3.8-fold greater MFE than ALDH^−^ cells (p = 0.001) (Figure 1C). Next, we investigated the *in vivo* tumor-initiating capabilities of ALDH^+^ cells isolated from the ER^+^ cell line MCF-7 following 6-day *in vitro* treatment with the AEs tamoxifen or fulvestrant (Figure 1D). Injection of 1,000 ALDH^+^ cells consistently gave rise to bigger tumors compared with the same number of ALDH^−^ cells, significantly so in tamoxifen- and fulvestrant-treated cells (Figure 1E). Extreme limiting dilution analysis revealed that on average the number of tumor-initiating cells was 4.2-fold higher in ALDH^+^ compared with the non-BCSC ALDH^−^ cells in all three conditions tested (Figure 1F). As few as 100 ALDH^+^ cells gave rise to tumors in mice whereas 100 ALDH^−^ cells failed to do so. These results highlight the increased tumor-initiating capabilities of the ALDH^+^ population in comparison with ALDH^−^ cells, implying the need to characterize this population of CSCs that survive AE therapies.

### Transcriptomic Characterization of ALDH^+^ Cells in Therapy-Resistant Patient Samples

**Figure 2:**
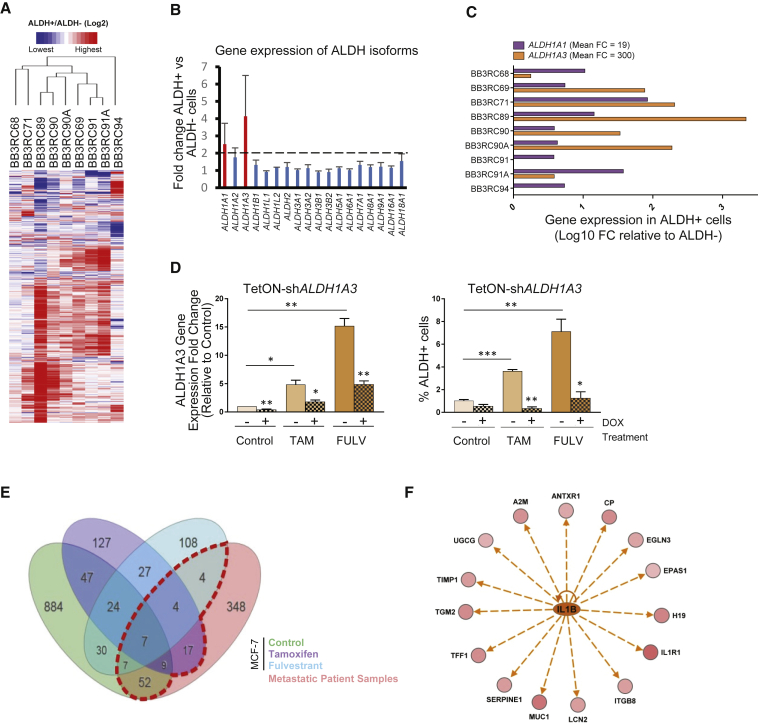
ALDH^+^ Cells from ER^+^ Metastatic Samples Show a Distinct Gene Expression Pattern Compared with ALDH^−^ Cells (A) Heatmap illustrating the 599 differentially expressed genes (447 up, 152 down) between ALDH^+^ and ALDH^−^ cells (red color shows gene upregulation, green shows downregulation in ALDH^+^ relative to ALDH^−^ cells identified by pairwise rank products with a threshold probability of false positives <0.05) from metastatic ER^+^ patient BCs. (B) Gene expression fold change (FC) between ALDH^+^ and ALDH^−^ cells of 18 ALDH isoforms detected in the Affymetrix array data. Mean FC for all metastatic samples (n = 9) is represented for each isoform. Red bar indicates isoforms with FC higher than 2. (C) qPCR analysis of *ALDH1A1* and *ALDH1A3* gene expression in the nine patient metastatic samples that were used in the Affymetrix array. Data are shown as log_10_ FC between ALDH^+^ and ALDH^−^ cells. Mean linear FC of the two ALDH isoforms for all samples is shown. (D) A stably transduced inducible sh*ALDH1A3* MCF-7 cell line was treated with control, tamoxifen (TAM), or fulvestrant (FULV) for 6 days concomitantly with (filled pattern) or without (solid bars) doxycycline (DOX). *ALDH1A3* mRNA levels were examined by qPCR (left) and percentage of ALDH^+^ cells was assessed using the Aldefluor assay (right). Data of at least three independent experiments are shown (^∗^p < 0.05, ^∗∗^p < 0.01, ^∗∗∗^p < 0.001). (E) Venn diagram illustrates meta-analysis of the MCF-7 cell line (control, tamoxifen, fulvestrant-treated ALDH^+^ versus ALDH^−^ cells) and the patient Affymetrix data (ALDH^+^ versus ALDH^−^ cells). iPathway guide software tool (AdvaitaBio) was used to plot the diagrams. The red dashed-line box indicates the 100 genes that are commonly differentially expressed in ALDH^+^ cells of patient samples and MCF-7 cell line. The log_2_ FC cutoff applied to the ALDH^+^ versus ALDH^−^ cells obtained from the meta-analysis data was 0.6. (F) Ingenuity Pathway Analysis diagram showing that IL-1β signaling is predicted to be activated (orange color) in the ALDH^+^ cell population. Straight arrows indicate network of 15 genes, predicted to be regulated by IL-1β, that were upregulated (in red) in ALDH^+^ cells. See also Figure S2.

To better understand the development of resistance to AE therapies in ER^+^ BC patients, we interrogated and compared the gene expression pattern between ALDH^+^ and ALDH^−^ cells in nine ER^+^ metastatic samples (Figure 2A). All patients had progressive disease since they required pleural effusion or ascitic drainage as palliative care, but while six samples were treated with endocrine therapies the other three were endocrine therapy-naive (Table S1). Overall, 599 genes were found to be differentially expressed (p ≤ 0.05) between the two ALDH cell populations among the 18,752 genes with measured expression (Table S2).

To identify which isoforms of ALDH are responsible for the Aldefluor activity of ALDH^+^ cells in metastatic ER^+^ patient samples, we investigated the mRNA expression levels of the 18 detected ALDH isoforms in our patient sample dataset. *ALDH1A1* and *ALDH1A3* showed the greatest fold change (FC) between ALDH^+^ and ALDH^−^ cells with a mean FC higher than 2 (Figure 2B). Validation by qRT-PCR confirmed the elevated expression of *ALDH1A1* and *ALDH1A3* isoforms in the ALDH^+^ compared with ALDH^−^ population, with a considerably higher averaged linear FC of *ALDH1A3* (300-fold) than *ALDH1A1* (19-fold) across the nine patient samples (Figure 2C). Interestingly, we also found that 6 days of AE treatment significantly upregulated *ALDH1A3* mRNA levels in two ER^+^ cell lines, MCF-7 and T47D (Figure S2A). Therefore, we used a doxycycline-inducible short-hairpin RNA system to test the effects of *ALDH1A3* silencing on AE resistance. *ALDH1A3* was stably downregulated by 58% compared with transfected cells not exposed to doxycycline, and there was a significant decrease in the induction of *ALDH1A3* mRNA levels following AE treatment in the knockdown (KD) cells (Figure 2D, left). The enrichment in the ALDH^+^ cell population after tamoxifen and fulvestrant treatments was significantly reduced in the ALDH1A3 KD cells (Figure 2D, right), indicating the importance of the ALDH1A3 isoform in ALDH^+^ cell population after AE therapy.

We also interrogated the gene expression profile of ALDH^+^ and ALDH^−^ populations from AE-treated MCF-7 cells. The meta-analysis from the patient and cell line microarray datasets (FC ≥ ±1.5 and p ≤ 0.05) revealed 100 genes commonly shared between ALDH^+^ cells of patient samples and ALDH^+^ cells of the MCF-7 cell line (Figure 2E and Table S3). Ingenuity Pathway Analysis for these genes predicted activation of eight upstream regulators (*Z* score ≥2.5), including several cytokines; for example, interleukin-1β (IL-1β) (Figure S2B). Of the 100 genes identified in the ALDH^+^ cell population, 15 were predicted to be regulated by IL-1β and these were all upregulated in the ALDH^+^ cells, which is consistent with activation of IL-1β signaling (Figure 2F). This activation was more obvious in the ALDH^+^ cells of AE-resistant samples, since 14 out of the 15 IL-1β-regulated genes were expressed at lower levels in the ALDH^+^ cells of the endocrine therapy-naive samples (Figure S2C). One of these genes was interleukin-1 receptor type 1 (IL1R1), which binds and transmits the signal of both IL-1α and IL-1β.

### AE Treatment Selects for IL1R1-Expressing ALDH^+^ Cells

**Figure 3:**
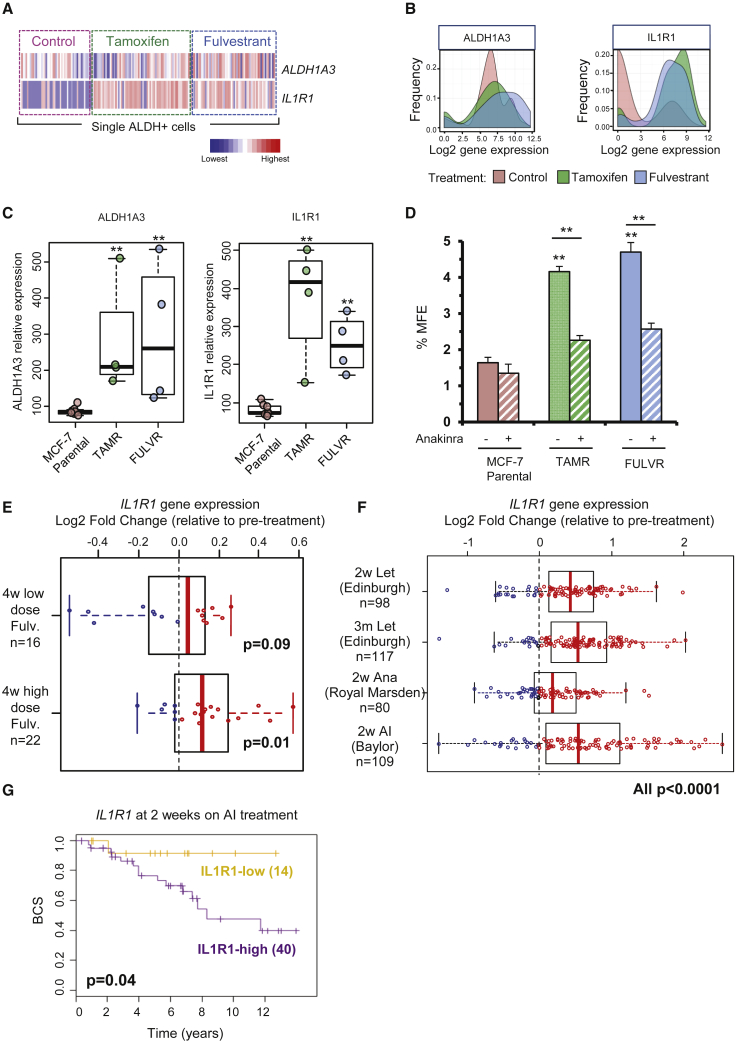
Single ALDH^+^ Cell Gene Expression in the MCF-7 Cell Line Identifies *IL1R1* Overexpression Following AE Treatment (A) Heatmap of the relative expression across single ALDH^+^ cells (columns) for *ALDH1A3* and *IL1R1* genes (rows). Cells are ordered by treatment, i.e., control (left), tamoxifen (middle), and fulvestrant (right). Colors represent expression levels from highest (red) to lowest (blue). (B) Density plots of gene expression in all single ALDH^+^ cells analyzed from the two different AE treatments and control. (C) Box plots and scatterplots show ALDH1A3 and IL1R1 relative gene expression from MCF-7 parental/unselected clonal sublines (n = 7) compared with tamoxifen-resistant (TAMR, n = 4) and fulvestrant-resistant (FULVR, n = 4) clonal sublines (GEO: GSE14986 dataset). Data from sublines grown without drugs (MCF-7 parental and TAMR) or with fulvestrant (FULVR). p value for at least four biological replicates calculated with Wilcoxon test, ^∗∗^p < 0.01. (D) MCF-7 parental, tamoxifen-resistant (TAMR), and fulvestrant-resistant (FULVR) cells were pre-treated in adherence with 10 μg/mL anakinra or vehicle control in the presence of 10 ng/mL IL-1β for 72 h. MFE was assessed after pre-treatments. Data are presented as mean ± SEM of three experiments with at least three technical replicates each. ^∗∗^p < 0.01. (E) Box plot and scatterplot show *IL1R1* expression from ER^+^ BC after pre-surgical 4-week treatment with fulvestrant (low-dose, 250 mg or high-dose, 500 mg) compared with *IL1R1* expression before treatment (Patani et al., 2014). Data are presented as log_2_ FC. Each patient sample is displayed as a blue (downregulation) or red (upregulation) circle. p value calculated with paired Wilcoxon test. (F) Box plot and scatterplot show *IL1R1* log_2_ FC gene expression in three different patient cohorts in response to 2 weeks (2w) or 3 months (3m) of letrozole (Let, Edinburgh dataset), anastrozole (Ana, Royal Marsden dataset), and AI (Baylor dataset) treatment compared with pre-treatment levels. Each patient sample is displayed as a blue (downregulation) or red (upregulation) circle. p value calculated with paired Wilcoxon test. (G) Kaplan-Meier curves represent BC specific-survival (BCS) for *IL1R1*-high and *IL1R1*-low of a cohort of 54 ER^+^ BC patients (Edinburgh) who received 2 weeks of AI treatment. p value is based on a log-rank test. See also Figure S3.

To study the effects of AE treatment on the ALDH^+^ population at the single-cell level, we analyzed the expression of *IL1R1* and *ALDH1A3* in 178 individual ALDH^+^ cells following tamoxifen or fulvestrant treatment. Sorted ALDH^+^ cells were injected and captured in the C1 system, followed by microscopic examination of cell singlets (Figure S3A). When comparing *IL1R1* gene expression profiles between control and AE-treated ALDH^+^ cells, we observed that gene expression levels of *IL1R1* increased significantly following tamoxifen and fulvestrant treatment (Figures 3A and 3B). In contrast, *ALDH1A3* expression was high in nearly all ALDH^+^ cells, with or without therapy, as expected (Figures 3A and 3B). *IL1R1* gene expression density plots revealed that control ALDH^+^ cells match a bimodal distribution with two distinct transcriptomic states: a population that comprises the majority of cells, which show none or very low *IL1R1* gene expression levels, and a small population of cells showing high *IL1R1* levels. However, following AE therapy the vast majority of ALDH^+^ cells show high *IL1R1* gene expression levels (Figures 3B and S3B). These results reveal the existence of cellular diversity within the ALDH^+^ population that can be unraveled by single-cell gene expression profiling, and highlight *IL1R1* as an important gene in AE-resistant BCSCs. Indeed, MCF-7 tamoxifen- and fulvestrant-resistant cell sublines express significantly higher levels of both *ALDH1A3* and *IL1R1* genes when compared with parental cell sublines (Figure 3C; Coser et al., 2009). In addition, AE-resistant MCF-7 cell lines show increased MFE when compared with the parental AE-sensitive cell line, which can be significantly reduced by anakinra, a recombinant form of human IL1R1 antagonist (Figure 3D).

To determine the clinical significance of identifying IL1R1 as facilitating AE resistance, we assessed *IL1R1* gene expression levels in patient breast tumors. Consistent with our cell line data, we found *IL1R1* gene expression levels to be increased in breast tumors following short-term administration of fulvestrant to patients (Figure 3E; Patani et al., 2014). In addition, we observed that *IL1R1* expression was significantly increased upon short- and long-term aromatase inhibitor (AI) treatment compared with baseline levels in four different patient cohorts totaling 404 patients (Dunbier et al., 2013; Ellis et al., 2017; Turnbull et al., 2015) (Figure 3F). Notably, we also found that elevated expression of *IL1R1* in ER^+^ patients who had been treated with AI for 2 weeks was significantly associated with a poor outcome (Figure 3G).

### Single-Cell RNA Profiling Identifies a Dormant ALDH^+^ Population that Is Expanded after AE Treatment

**Figure 4:**
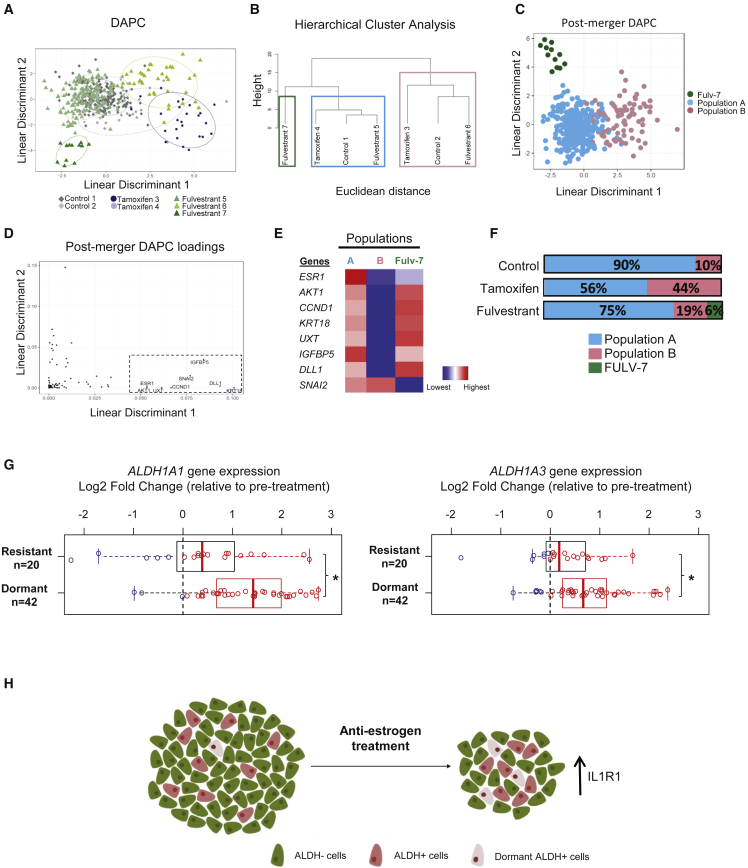
Single-Cell Gene Expression Data Reveal a Dormant ALDH^+^ Population (A) Scatterplot of the two first linear discriminants from discriminant analysis of DAPC analysis for 377 single ALDH^+^ MCF-7 cells using as classifier the clusters identified through Mclust. The scatterplot shows the cluster of individual ALDH^+^ cells (rhomboids: control group; circle: tamoxifen group; triangles: fulvestrant group). Control-treated ALDH^+^ cells (gray) clustered within two groups (clusters 1 and 2), Tamoxifen-treated ALDH^+^ cells (blue) also clustered within two groups (clusters 3 and 4), and fulvestrant-treated ALDH^+^ cells (green) clustered within three groups (clusters 5, 6, and 7). Pooled data of three independent experiments are shown. (B) Ward hierarchical clustering of cell clusters using Euclidean distance of all the genes. Boxes represent clusters with an unbiased p value of >0.90 indicating that these clusters are robust, thus identifying three groups of cells: two major ones, renamed as population A (blue box) and population B (pink box) and a smaller one corresponding to Fulvestrant-7 (green box). (C) Scatterplot of the DAPC analysis for single ALDH^+^ MCF-7 cells after treatment using as classifier the clusters identified in (B). Linear discriminant 1 accounts for most of the differences between population B and the other two. (D) Distribution of the gene importance to build linear discriminants 1 and 2. Genes above threshold 0.05 of linear discriminant 1 are labeled. (E) Heatmap of relative gene expression across the three ALDH^+^ populations identified (A, B, fulvestrant 7 [fulv-7]) for the eight most important genes in the separation between population B and the others. Colors represent expression levels from highest (red) to lowest (blue). (F) Bar charts show the percentage contribution of each ALDH^+^ subpopulation within the ALDH^+^ cells treated with control, tamoxifen, or fulvestrant. (G) Box plots and scatterplots show *ALDH1A1* and *ALDH1A3* expression from ER^+^ dormant and acquired resistant tumors after 4 months of neoadjuvant treatment with letrozole compared with expression before treatment (Selli et al., 2019). Data are presented as log_2_ FC. Each patient sample is displayed as a blue (downregulation) or red (upregulation) circle. p value calculated with paired Wilcoxon test. (H) Diagram showing that AE therapies do not target ALDH^+^ cells and enrich for a dormant IL1R1^+^ALDH^+^ cell population. See also Figure S4.

*IL1R1* and *ALDH1A3* single-cell gene expression revealed heterogeneity within ALDH^+^ cells; therefore, we decided to investigate the existence of putative subpopulations within the ALDH^+^ cell population. We examined the mRNA expression level of 68 genes across 377 single ALDH^+^ MCF-7 cells following control, tamoxifen, or fulvestrant treatment. The 68-gene list (see Supplemental Experimental Procedures) comprised key regulators associated with stemness, self-renewal pathways, and markers related to ALDH^+^ cells that were identified in the whole gene expression dataset (Figure 2). A Gaussian mixture model approach to estimate and assign clusters to the cells predicted the existence of seven different cellular ALDH^+^ populations (control: 1 and 2, tamoxifen: 3 and 4, fulvestrant: 5, 6, and 7) (Figure 4A). Some of these initial clusters were merged, based on their gene expression similarities using Ward's hierarchical clustering on Euclidean distance coupled with bootstrapping to estimate branch robustness. This analysis resulted in two major populations of cells, population A and population B, which were both made of clusters from the three different treatments, and a small population of fulvestrant-treated cells (fulvestrant 7) that were distinct from the rest of the cells (Figure 4B). Next, we applied discriminant analysis of principal components (DAPC) to create a graphical representation of these three distinct populations (Figure 4C). The eight genes most associated with the first linear discriminant had the highest contribution to the separation of population B from the other populations (Figure 4D). Genes associated with cell proliferation, for example the cycle regulator *CCND1* and protein kinase *AKT1*, were downregulated in population B compared with population A, whereas the expression of the mesenchymal marker *SNAI2* was higher in the former (Figure 4E). Moreover, population A, which comprised the vast majority of the ALDH^+^ cells analyzed (82%), exhibited higher expression of proliferative markers *PCNA* and *KI67* in comparison with population B (Figure S4A). Interestingly, only 10% of the non-treated ALDH^+^ cells belonged to the quiescent population B; however, following tamoxifen and fulvestrant treatment the percentage of quiescent cells represented 44% and 19% of the total cells, respectively (Figure 4F). A recent study (Selli et al., 2019) investigated gene expression changes of dormant and acquired resistant ER^+^ tumors treated with an AI for more than 4 months. Notably, *ALDH1A1* and *ALDH1A3* gene expression levels were significantly increased in dormant tumors compared with acquired resistant tumors, which supports the existence of an ALDH^+^ dormant population after AE treatment (Figure 4G). Relative to pre-treatment, the dormant tumors also had significantly increased expression of both *ALDH1A1* and *ALDH1A3* as well as *IL1R1* and *SNAI2*, along with reduced *CCND1* (Figure S4B), consistent with the results above for the dormant population identified by single-cell analysis. These data suggest that AE resistance can be driven by non-proliferative dormant ALDH^+^ cells and support a potential role for IL1R1-targeted therapy to overcome resistance in ER^+^ BCs (Figure 4H).

## Discussion

Previously, we reported that ALDH^+^ cells are resistant to AE therapy and that high ALDH1 expression predicts resistance in women treated with tamoxifen (Simões et al., 2015). Our findings here establish a role for the IL1R1 signaling pathway in the regulation of AE-resistant ALDH^+^ BCSCs. We also identify heterogeneity in the ALDH^+^ cell population and an expansion of a quiescent ALDH^+^ subpopulation after AE therapies.

Firstly, we showed that BC cells contain a population of ALDH^+^ cells that survive AE treatments, which maintain higher mammosphere-forming and tumor-initiating cell frequency than ALDH^−^ cells. We next wanted to further characterize these cells and the mechanisms that drive them. ALDH1A1 and ALDH1A3 isoforms are both reported to be predictive biomarkers of poor clinical outcome in BC (Liu et al., 2014; Marcato et al., 2011), and we found them to be the most highly increased among 18 ALDH isoforms detected in metastatic patient-derived ALDH^+^ BC cells. ALDH1A3 KD confirmed that this isoform is crucial for enriching the ALDH^+^ population following AE treatment. These data support the growing body of literature describing the involvement of ALDH1A3 in cancer stemness, tumor progression, and poor prognosis.

We found that ALDH^+^ cells have a different gene expression profile compared with ALDH^−^ cells in both ER^+^ metastatic patient samples and MCF-7 cells. In particular, genes that predicted activation of pro-inflammatory cytokine IL-1β signaling, including *IL1R1*, were expressed at higher levels in ALDH^+^ cells. Furthermore, these genes were expressed at even higher levels in ALDH^+^ cells of AE-treated compared with AE-naive patient samples. Gene expression analysis of ALDH^+^ cells from AE-sensitive primary BC samples would validate our findings further but was not possible in the present study. By using single-cell gene expression profiling in the ALDH^+^ cell population, we confirmed *IL1R1* to be significantly upregulated in AE-treated ALDH^+^ cells compared with control cells. Moreover, AE-resistant cell lines express higher levels of *IL1R1* and display enriched CSC activity that is mainly dependent on IL-1β signaling, since it is significantly reduced by IL1R1 inhibition. Importantly, we found that expression of *IL1R1* is induced in the tumors of patients treated with AE therapies and predicts treatment failure. These data indicate that IL-1β signaling is likely to be important for CSCs to drive AE resistance in BC. *IL1β* expression correlates with increased aggressiveness and enhanced metastatic potential of BC cells, suggesting IL-1β as a potential biomarker for predicting which patients are likely to be diagnosed with BC metastasis, specifically to bone (Tulotta et al., 2019). Indeed, our group has recently demonstrated the importance of IL-1β-IL1R signaling in regulating stem cell activity in BC metastasis to the bone (Eyre et al., 2019). We established that bone marrow-derived IL-1β stimulates breast CSC colonization in the bone by inducing intracellular nuclear factor κB and Wnt signaling in breast CSCs. These findings suggest that metastatic dissemination is selecting for IL1R^+^ CSCs that colonize the IL-1β-producing bone marrow.

Single-cell targeted transcriptome analysis revealed the existence of distinct clusters within ALDH^+^ cells and the expansion of a quiescent ALDH^+^ population (population B) after AE therapies. Heterogeneity within BCSCs of MCF-7 cells has previously been described using different CSC functional assays, such as mammospheres, growth in hypoxia, and PKH26 retention, to isolate single cells for gene expression analysis (Akrap et al., 2016). Our data suggest that population B represents a small population of non-dividing quiescent ALDH^+^ cells that survive AE treatments, which may enable them to survive for long periods of time and eventually lead to late recurrence in ER^+^ BC patients. This idea is supported by data from AI-induced dormant tumors that express increased levels of *ALDH1A1* and *ALDH1A3* genes. Recently, single-cell RNA profiling of normal breast samples identified four cell clusters within the ALDH^+^ cell population (Colacino et al., 2018). Interestingly, population B resembles cluster 3 identified in this publication, which was characterized by high expression of mesenchymal markers, including *SNAI2*, and low expression of proliferative genes, such as *KI67*, *PCNA*, and *CCND1*. Population B expresses low levels of *AKT1*, and AKT1^low^ cancer cells have been reported to be quiescent cells that survive chemotherapy in breast tumors (Kabraji et al., 2017).

Combination therapies targeting both bulk tumor cells and BCSCs should reduce the probability of tumor relapse; therefore, pharmacological inhibitors that target CSC pathways have been highly pursued and are being tested in patients (Brooks et al., 2015). In our model, both proliferative and dormant AE-resistant BCSCs express *IL1R1*. This suggests that anti-IL1R1 therapies, such as anakinra or canakinumab (human anti-IL-1β monoclonal antibody), could represent a new strategy to target AE-resistant CSCs.

In conclusion, the present work contributes to our understanding of the cellular heterogeneity present in the AE-resistant BCSC population. Our work suggests that CSC dormancy is an adaptive strategy to evade AE treatments and supports the targeting of ALDH^+^IL1R1^+^ cells to reverse AE resistance. This work highlights the advantages of single-cell transcriptomic analysis, rather than bulk tissue, to interrogate the cellular heterogeneity within the ALDH^+^ CSC population. Further understanding of the dormant ALDH^+^ population that survives AE therapies, particularly using clinical samples, will provide new insights for prevention and treatment of recurrences of ER^+^ BC.

## Experimental Procedures

A comprehensive description of the methodology is included in Supplemental Information.

### Breast Cancer Samples

Consented, de-identified pleural effusion or ascitic fluids were collected at the Christie NHS Foundation Trust (UK) or the University of Michigan (USA). The clinicopathological details of the samples are shown in Table S4.

### ALDH^+/−^ Cell Isolation

BC cells were stained using the Aldefluor assay (STEMCELL Technologies) following the manufacturer's protocol and isolated using the Influx cell sorter (BD Biosciences).

### Single-Cell Capture and Transcriptomics Profiling

Single ALDH^+^ MCF-7 cells were captured within the C1 system using the medium C1 Single-Cell Preamp Integrated Fluidic Circuit (IFC, 10–17 μm) chips (Fluidigm, 100-5480). Individual cells were visualized using the Leica Widefield Low Light microscope. Cell loading, lysis, reverse transcription, and cDNA pre-amplification were performed within the C1 system following the manufacturer's instructions. We undertook three independent experiments that resulted in the single-cell transcriptomics profiling of 377 single cells. Data were acquired using the 96.96 Dynamic Array IFC Biomark chips (Biomark HD Real-Time PCR System, Fluidigm) to interrogate the expression of 68 TaqMan assays in each cell. Data analyses included different quality control steps and two iterative runs of clustering to identify cell populations. Further details on experimental design and data processing are described in the Single-Cell Data Analysis section of Supplemental Experimental Procedures.

### Data and Code Availability

The Affymetrix data have been deposited in NCBI's Gene Expression Omnibus repository under series accession number GEO: GSE136287.

## Author Contributions

B.M.S. and R.B.C. conceptualized the study. A.S.-C., R.B.C., and B.M.S. designed and carried out the experiments, performed data interpretation, and wrote the manuscript. E.C.-G. carried out bioinformatics single-cell analysis and wrote parts of the manuscript. A.H.S. performed bioinformatics analysis on Affymetrix data and patient data. M.I.J. created the knockdown cell line. C.C. and M.D.B. helped carry out single-cell experiments. M.E.B. provided patient samples from the Michigan cohort. N.J.H. performed mammosphere assays with cell lines. A.S.-G., R.E., M.S.W., and S.J.H. advised on experimental design and revised the manuscript. All authors edited and approved the final version.

## Supplemental Information

Document S1. Supplemental Experimental Procedures, Figures S1–S4, and Table S4

Table S1. Timeline of Anti-estrogen Therapies Received by Each Patient Prior to Sample Collection, Related to Figure 2

Table S2. List of Genes Differentially Expressed Between ALDH^+^ and ALDH^−^ Cell Populations in Patient Samples, Related to Figure 2

Table S3. List of Differentially Expressed Genes Commonly Shared Between ALDH^+^ Cells of Patient Samples and ALDH^+^ Cells of the MCF-7 Cell Line, Related to Figure 2

Document S2. Article plus Supplemental Information

## Footnotes

Supplemental Information can be found online at https://doi.org/10.1016/j.stemcr.2020.06.020.
