# Supplementary material for: Increased Expression of Interleukin-1 Receptor Characterizes Anti-estrogen-Resistant ALDH+ Breast Cancer Stem Cells
Source: Stem Cell Reports. 2020 Jul 23;15(2):307–16. doi: 10.1016/j.stemcr.2020.06.020 (PMC7419713; doi:10.1016/j.stemcr.2020.06.020)
Supplement: Document S1. Supplemental Experimental Procedures, Figures S1–S4, and Table S4 [file mmc1.pdf]

Stem Cell Reports, Volume *15*

Supplemental Information

Increased Expression of Interleukin-1 Receptor Characterizes Anti-estrogen-Resistant ALDH+ Breast Cancer Stem Cells

Aida Sarmiento-Castro, Eva Caamaño-Gutiérrez, Andrew H. Sims, Nathan J. Hull, Mark I. James, Angélica Santiago-Gómez, Rachel Eyre, Christopher Clark, Martha E. Brown, Michael D. Brooks, Max S. Wicha, Sacha J. Howell, Robert B. Clarke, and Bruno M. Simões

Supplementary Figure 1, Related to Figure 1

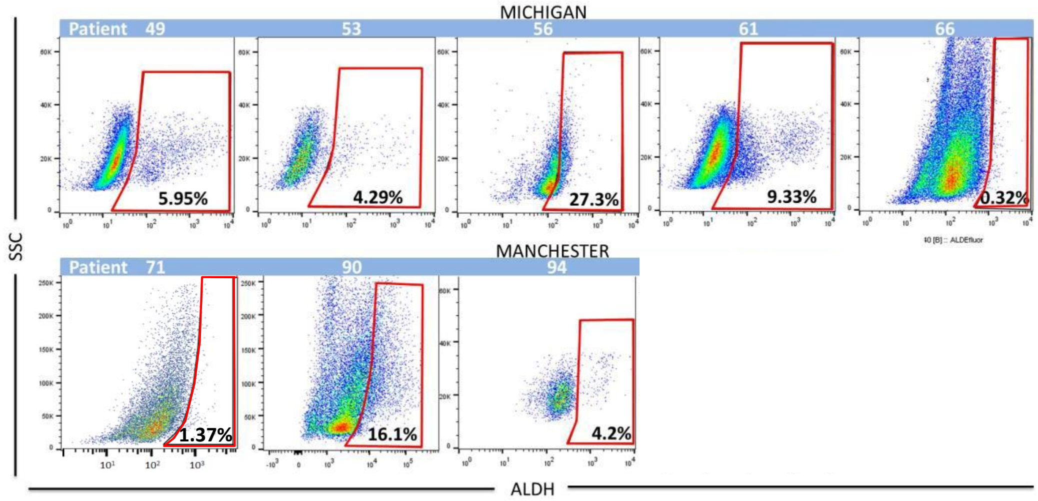

Figure S1. FACS plots showing percentage of ALDH+ cells, measured by the Aldefluor assay, in metastatic patient samples. ALDH+ cells (red box) from Michigan's biobank (top) and Manchester's biobank (bottom) patient-derived samples are shown.

Supplementary Figure 2, Related to Figure 2

A

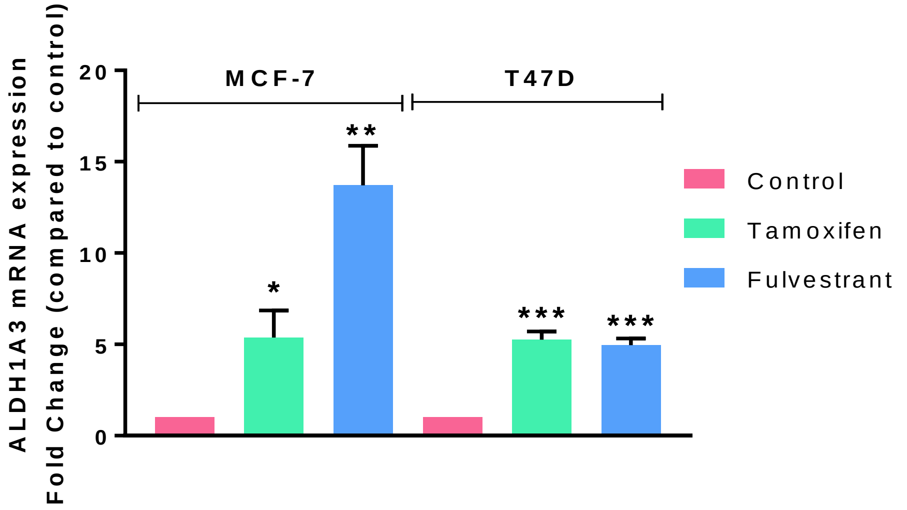

B

Upstream Regulator	Predicted Activation State	Activation z-score	p-value of overlap
TGFB1	Activated	3.29	0.00000468
AGT	Activated	3.04	0.0000123
SMARCA4	Activated	2.967	2.9E-09
IL6	Activated	2.925	0.00000036
TNF	Activated	2.875	7.56E-13
IL1B	Activated	2.674	0.000000434
OSM	Activated	2.545	0.0000608
ERK	Activated	2.53	4.18E-08

C

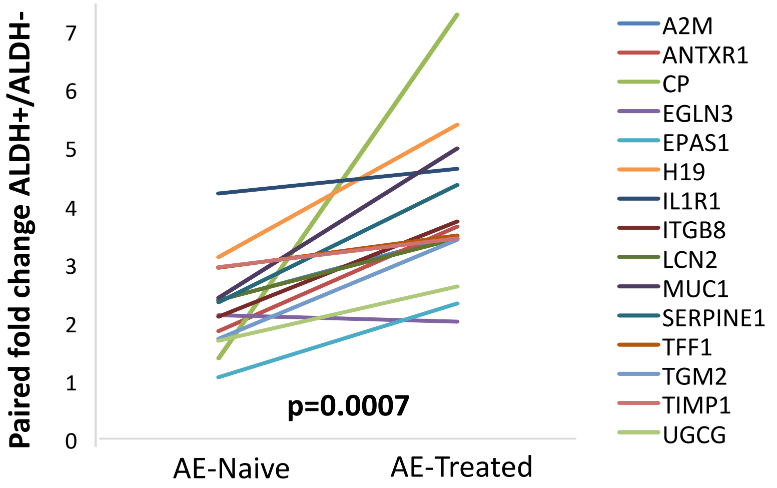

Figure S2. A) ALDH1A3 mRNA expression in MCF-7 and T47D cells following tamoxifen (green) and fulvestrant (blue) treatment compared to control (pink). Data of at least 3 independent experiments are shown (*p<0.05, **p<0.01, ***p<0.001). **B)** List of upstream regulators and respective predicted activation (with z-score ≥2.5) identified by Ingenuity Pathway Analysis (IPA) of 100 genes commonly expressed in ALDH+ cells of patient samples and MCF-7 cells. **C)** Gene expression of the 15 genes that predict IL1β activation in the ALDH+ cells of the 6 AE-treated metastatic samples (BB3RC68, BB3RC69, BB3RC71, BB3RC89, BB3RC91, BB3RC91A) and in the ALDH+ cells of the 3 AE-naïve metastatic samples (BB3RC90, BB3RC90A, BB3RC94). Graph shows the average paired fold change in ALDH+ vs ALDH- in both groups. P-value calculated with paired t-test comparing AE-treated with AE-naïve samples.

Supplementary Figure 3, Related to Figure 3

A

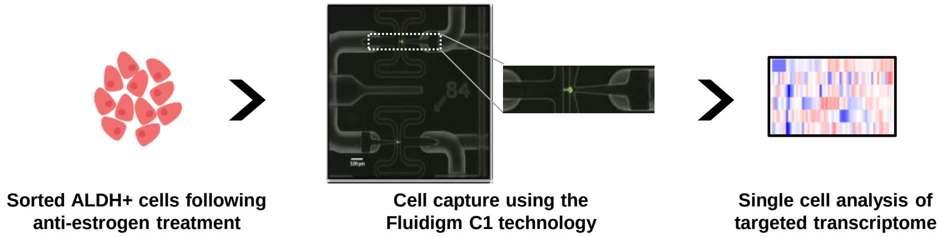

B

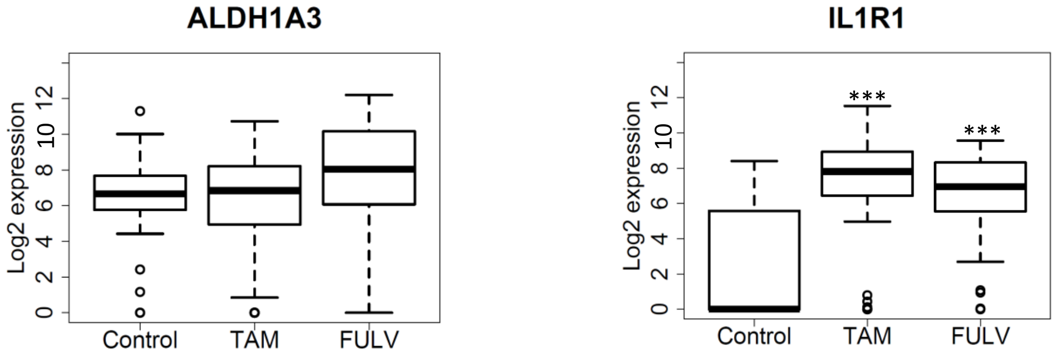

Figure S3. A) Schematic overview of the experimental approach to profile single ALDH+ cells. MCF-7 cells treated with either tamoxifen, fulvestrant or control were sorted into single cells and transcription profiles of genes of interest were obtained and analysed as described in the methods. **B)** Boxplots of *ALDH1A3* and *IL1R1* gene expression in cells treated with vehicle (Control), tamoxifen (TAM) and fulvestrant (FULV). Log2 expression distribution in all the cells is shown as boxes containing the interquartile ratio (first and third quartiles) with the median (bold line) and whiskers representing the 5–95% range. Kruskal-Wallis with Dunn's post-hoc correction was used to compare tamoxifen/fulvestrant treated cells versus control cells.***Pvalue<0.001

Supplementary Figure 4, Related to Figure 4

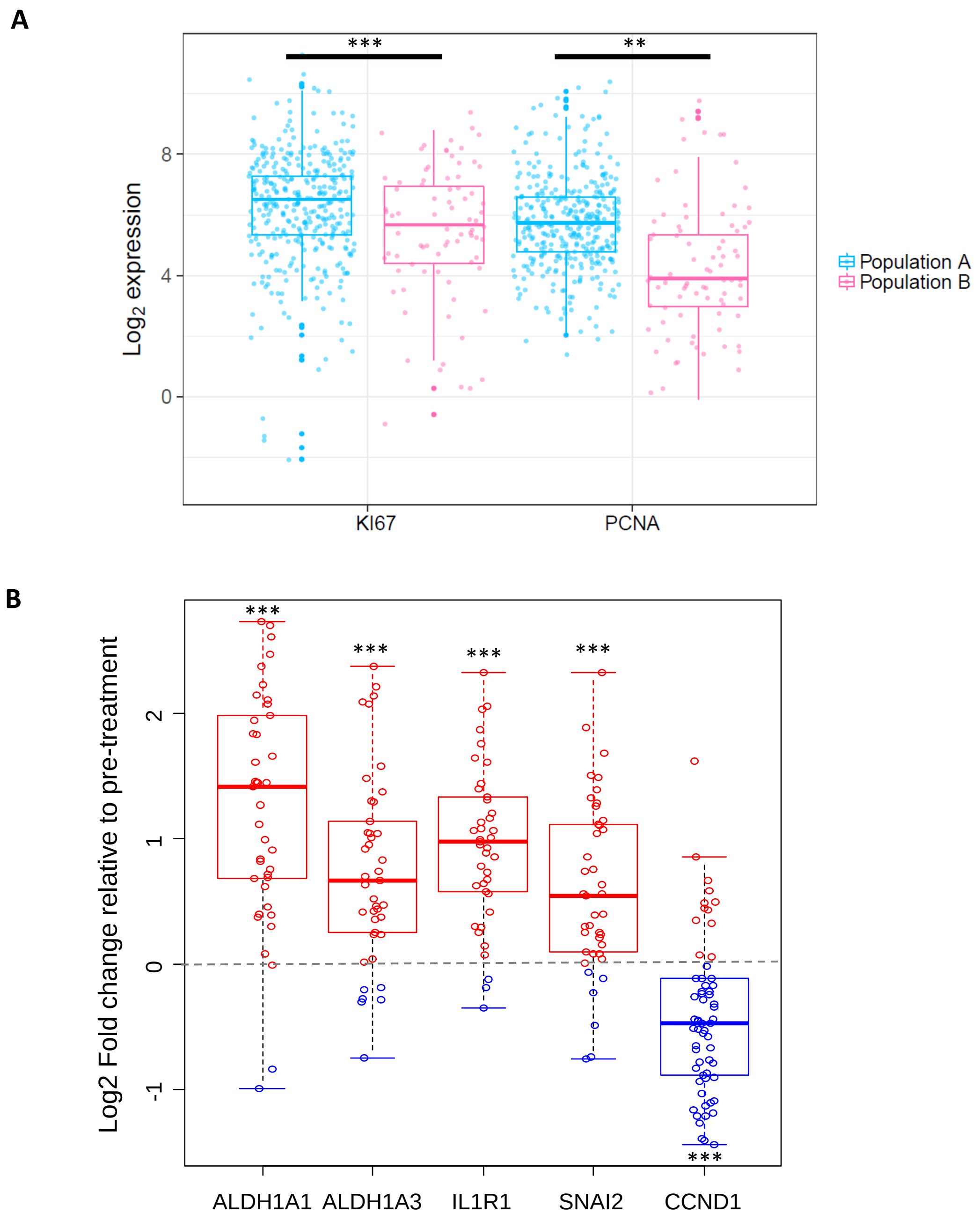

Figure S4. A) Boxplots of *Ki67* and *PCNA* gene expression in cells of Population A and B. Log2 expression distribution in all the cells is shown as boxes containing the interquartile ratio (first and third quartiles) with the median (bold line) and whiskers representing the 5–95% range. Each point within the plot represents a cell for the gene specified. Mann-Whitney U Test was used to compare Population B vs Population A. P values were adjusted for FDR with Benjamini and Hochberg method. (***Adj -Pvalue <0.001; **Adj-Pvalue<0.01). **B)** Boxplots show *ALDH1A1*, *ALDH1A3*, *IL1R1*, *SNAI2, CCND1* gene expression from ER+ dormant tumours after 4-months neoadjuvant treatment with letrozole compared to expression before treatment (Selli et al., 2019). Data is represented as Log2 fold change. Each patient sample is displayed as a blue (down-regulation) or red (up-regulation) circle. P-value calculated with paired Wilcoxon test.

Supplementary Tables

Table S1, Related to Figure 2. Timeline of anti-estrogen therapies received by each patient prior to sample collection.
Please refer to Supplemental spreadsheet file.

Table S2, Related to Figure 2. List of genes differentially expressed between ALDH+ and ALDH- cell populations in patient samples.
Please refer to Supplemental spreadsheet file.

Table S3, Related to Figure 2. List of differentially expressed genes commonly shared between ALDH+ cells of patient samples and ALDH+ cells of the MCF-7 cell line.
Please refer to Supplemental spreadsheet file.

Table S4, Related to Figure 1 and Figure 2. Clinico-pathological information of the Michigan and Manchester patient datasets. QS: Quick score.

Patient	ER	PR	Chemotherapy	Endocrine Therapy	Bone Therapy	Other Therapy	Metastasis
**Mi49**	+	+	Capecitabine<br>Cyclophosphamide<br>Doxorubicin<br>Eribulin<br>Etoposide<br>Gemcitabine<br>Ixabepilone<br>Taxol	Anastrozole<br>Fulvestrant<br>Goserelin	Zometa<br>Zolendronic acid	Anti-DLL4 antibody: OMP-21M18	Bone<br>Brain<br>Pleura
**Mi53**	+	+	Cyclophosphamide<br>Doxorubicin<br>Taxol	Anastrozole<br>Tamoxifen	Zometa	-	Bone<br>Liver
**Mi56**	+	+	Capecitabine<br>Carboplatinum<br>Paclitaxel<br>Vinorelbine	Exemestane<br>Letrazole<br>Tamoxifen	Denosumab	-	Bone<br>Chest wall<br>Lymph node
**Mi61**	+	+	Cyclophosphamide<br>Docetaxel<br>Doxorubicin	Anastrozole<br>Exemestane	Denosumab	-	Bone<br>Pleura
**Mi66**	+	+	Capecitabine<br>Carboplatin<br>Gemcitabine	Arimidex<br>Tamoxifen	Denosumab	-	
**BB3RC68**	QS8		Capecitabine<br>Fluorouracil + Epirubicin + Cyclophosphamide (FEC)	Tamoxifen<br>Anastrazole<br>Fulvestrant	-	-	Bladder<br>Liver<br>Lung<br>Lymph nodes<br>Peritoneum
**BB3RC69**	96%	77%	-	Tamoxifen<br>Letrozole<br>Anastrazole	Pamidronate<br>Zoledronic Acid	-	Bone<br>Lymph node<br>Peritoneum
**BB3RC71**	54%	72%	Capecitabine<br>Eribulin<br>FEC<br>Taxol<br>Taxotere<br>Vinorelbine	Tamoxifen<br>Anastrazole<br>Fulvestrant<br>Exemestane	Pamidronate<br>Zoledronic Acid<br>Ibandronic Acid	Herceptin<br>Lapatinib	Bone<br>Liver<br>Pleura
**BB3RC89**	QS 8	QS 8	Capecitabine<br>FEC<br>Taxol	Tamoxifen<br>Letrozole<br>Exemestane	-	-	Bone<br>Liver<br>Lung
**BB3RC90-90A**	QS 8	QS 8	Capecitabine	-	-	-	Bone<br>Liver<br>Pleura
**BB3RC91-91A**	96%	98%	Docetaxel<br>FEC	Tamoxifen<br>Letrozole<br>Anastrazole	-	Everolimus	Bone<br>Liver<br>Omentum<br>Peritoneum
**BB3RC94**	+	+	Treatment naïve				Omentum<br>Peritoneum

N.B. Samples 90 and 90A are from the same patient but were taken at different time points. The same applies to samples 91 and 91A.

SUPPLEMENTAL EXPERIMENTAL PROCEDURES

Breast cancer samples

Metastatic fluids were collected at the Christie NHS Foundation Trust (UK) in accordance with local research ethics committee guidelines (study number: 05/Q1402/25) or the University of Michigan (study number: IRBMED 2001-0344/HUM00042204). Fluids were spun at 1000 g for 10 min at 4ºC and pellets were resuspended in Phosphate Buffered Saline (PBS). Erythrocytes and leucocytes were depleted from the metastatic fluids by using density gradient Lymphoprep (Stemcell Technologies) following manufacturer's protocol. Clinical information about patient samples is shown in **Table S4**.

Breast cancer cell lines

MCF-7 parental, Tamoxifen- and Fulvestrant-resistant cell lines were a kind gift from Dr Julia Gee (University of Cardiff, Wales) and were cultured as previously reported (Simões et al., 2015). Cell lines were grown in monolayer in the presence of 10 ng/ml recombinant human IL1β (201-LB, R&D systems) and treated with 10 µg/ml Anakinra (Amgen, Cambridge, UK) or vehicle for 72 Hours prior to plating in mammosphere culture.

ALDH+/- cell isolation

Breast cancer cells were re-suspended in Aldefluor buffer and incubated in the presence of the Aldefluor reagent bodipyaminoacetaldehyde (BAAA) (Aldefluor assay, Stemcell Technologies) for 40 minutes at 37ºC, following the manufacturer's protocol. A subset of cells was also incubated with the selective ALDH inhibitor diethylaminobenzaldehyde (DEAB) to distinguish between

ALDH+ and ALDH- cells. When performing single-cell experiments using the C1 system (Fluidigm), cells were stained for CD44 (CD44-APC; BD,) and CD24 (CD24-PECY7) expression as well as ALDH activity in order to isolate ALDH+ cells that are not CD44^{high} CD24^{low}. Following incubation, cells were washed with PBS and stained with the cell viability dye 7-aminoactinomycin (7AAD, BD). Cells were then FACS-sorted into 200 µl of 2% Fetal Bovine Serum in Hank's Balanced Salt Solution (HBSS) using the InFlux (BD bioscience). Single colour stains were included for compensation and gating purposes. Data was analysed using FlowJo 10.1.

Mammosphere culture

Cells from primary samples were seeded at a density of 500 cells/cm^2 in 6-well polyHEMA (Poly (2-hydroxyethylmethacrylate)) coated plates containing mammosphere media (DMEM/F12 media with L-Glutamine (Gibco), B27 supplement (Gibco; 12587) and 20 ng/ml EGF (Sigma)). Similarly, MCF-7 cells were seeded at a density of 200 cells/cm^2. Cells were cultured for 7 days (primary samples) or 5 days (cell lines) at 37ºC before counting mammospheres greater than 50 µm. Mammosphere forming efficiency (MFE) was calculated by dividing the number of mammospheres by the number of cells seeded per well and is expressed as the mean percentage of MFE (Shaw et al., 2012). For patient samples, experiments were carried out with at least 3 technical replicates (where possible, depending on the number of cells available after FACS-sorting). For cell line experiments, each experiment represents at least 3 technical replicates and three biological repeats.

Transplantation assays

In vivo studies were carried out in accordance with the UK Home Office (Scientific Procedures) Act 1986 under project licence PPL40/3645 and study protocols were approved by the CRUK Manchester Institute Animal Welfare and Ethical Research Board (AWERB).

MCF-7 cells were treated *in vitro* with 10^{-6} M 4-Hydroxytamoxifen (Sigma-Aldrich, H7904), 10^{-7} M fulvestrant (ICI 182,780, Tocris, 1047) or ethanol (vehicle) for 6 days following staining with the Aldefluor assay. Serial limiting dilution of sorted ALDH+ and ALDH- cells (10,000; 1,000; 100 cells) were resuspended in mammosphere media mixed 1:1 with Matrigel (BD bioscience, 356234) and inoculated subcutaneously into the left and right flanks of female NOD/SCID Gamma (NSG) mice. All in vivo work was carried out using n=4 mice for each condition. 90-day slow release estrogen pellets were implanted subcutaneously into mice before cell injection (0.72 mg, Innovative Research of America) and, after day 90, 8 µg/ml of 17-beta estradiol was administered in drinking water. Tumour measurements were taken three times a week and tumour size was calculated using the formula:

$$\text{Tumour size} = 0.5 \times \text{Length} \times \text{Width}^2$$

Positive tumour growth was assessed at week 20 after cell injection by determining the mice bearing a tumour greater than 300 mm^3. Extreme Limiting Dilution Analysis (ELDA) was performed using software available at http://bioinf.wehi.edu.au/software/elda/ (The Walter and Eliza Hall Institute of Medical Research) to assess differences in stem cell frequency.

RNA extraction and Real-Time PCR

ALDH+ bulk cells (≥10,000 cells) were sorted into 100 μl of lysis buffer containing 1% β-Mercaptoethanol, following by cell disruption and homogenisation via vortexing for 1 minute. RNA was extracted using the RNeasy Plus Micro Kit (Qiagen, 74034) with on-column DNase treatment following manufacturer's protocol. The Bioanalyzer (Agilent 2100 Bioanalyzer system, Agilent Technologies) and the Qubit (Thermofisher Scientific) were used for quantitation and quality control of the RNA.

Bulk transcriptome analysis

Human Array Gene 1.0 ST (Affymetrix) GeneChips were used to assess mRNA expression profile in bulk ALDH+ and ALDH- cells. Double stranded amplified cDNA was generated using the Ovation Pico WTA System V2 (NuGen) and the Single Primer Isothermal Amplification (SPIA) following manufacturer's guidelines. cDNA was fragmented and labelled prior hybridisation onto the array (GeneChip hybridization Oven 640, Affymetrix). The GeneChip array was then washed and stained using the Fluidics Station protocol FS450_0007 and the Affymetrix GeneChip Command Console Software (Affymetrix) following manufacturer‘s guidelines. The GeneChip array was scanned using the Scanner 3000 system with autoloader (Affymetrix).

Microarray data from cell line and patient samples were processed using the *Affy* package in R (Gautier et al., 2004). Data was quantile-normalised and Log2 transformed. Differential gene expression analysis was carried using paired Rank Products (Breitling et al., 2004). Meta-analysis was performed using iPathwayGuide (AdvaitaBio). Statistical significance for RNA expression was assessed using t-test parametric testing.

Single-cell data analysis

The following 68 genes were used for single-cell targeted transcriptome analysis.

ABCG2	*CDH3*	*FBXW7*	*IGFBP5*	*LIN28A*	*NOTCH3*	*TAZ*
AKT1	*CTNNB1*	*GAPDH*	*IL1R1*	*MET*	*NUMB*	*TGFB1*
ALDH1A3	*CXCR1*	*GATA3*	*IL6R*	*MKI67*	*PCNA*	*TGFBR1*
AR	*CXCR4*	*GJA1*	*IL6ST*	*MKP1*	*PGR*	*TP53*
BRCA1	*CYR61*	*GPRC5A*	*ITGA6*	*MTOR*	*PIK3CA*	*TSPAN6*
CA12	*DLL1*	*GSK3B*	*JAG1*	*MUC1*	*POU5F1*	*TWIST1*
CCND1	*ENAH*	*HER2*	*JAG2*	*NANOG*	*RAB7A*	*UXT*
CD24	*EPCAM*	*HES1*	*KRT18*	*NFKB1*	*SNAI2*	*YAP1*
CD44	*ESR1*	*HPRT1*	*KRT19*	*NOTCH1*	*SOCS3*	
CDH1	*EZH2*	*ID1*	*KRT8*	*NOTCH2*	*SOX2*	

Data generated by the Biomark (Fluidigm) were converted into Log2 expression values and quality controls were undertaken. These included data filtering to remove all values under the limit of detection, which was set to threshold cycles (Ct) greater than 28; the removal of genes expressed in 3 or less cells within each treatment and, the removal of outliers (via the function identifyOutliers implemented in the R package FluidigmSC - Fluidigm Corporation, 2014). Missing completely at random values were estimated and inputted using the R package MICE (Azur et al., 2011). Principal Component Analysis revealed a batch effect between experiments that was corrected using ComBAT implemented within the sva package in R (Leek et al., 2019). We undertook a statistical approach to eliminate doublets derived from equipment unfitness (Fluidigm Corporation, 2016). Using the package Mclust in R we fitted Gaussian mixture models to identify cell clusters within each treatment. These models indicated the existence of two very well defined cell clusters in each condition. The nature of these clusters was further investigated by plotting the average Log2 expression per gene in both clusters, pointing towards a stratification into

doublets and singlets. Clusters corresponding to singlets were taken forward for the analysis. This corresponds to 444 cell signals from 377 cells, proportions shown in table below. 96% of replicate analyses assigned cells to the same population (population A, population B or fulvestrant 7).

Experiment	Untreated cells	Tamoxifen treated cells	Fulvestrant treated cells
1	35	9	49
2	96	28	80
3	34	11	35
3 – Technical Replicates	31	9	27

With the aim of identifying different cell populations within treatment we used a finite Gaussian mixture model to (a) estimate the number of clusters within the data (function Mclust within the R package Mclust (Scrucca et al., 2016)) and (b) generate those clusters. Ward hierarchical clustering with bootstrapping (Ward, 1963) was undertaken with the package pvclust in R to find similarities between identified clusters and merge smaller clusters into larger ones (threshold of Approximately Unbiased (AU) p-value greater than 0.9). Merged clusters were assessed for biases regarding batch and plate and it was confirmed that they were not linked due to experimental handling. Further analysis of cluster similarities and genes associated to cluster differences were undertaken using Discriminant Analysis of Principal Components (DAPC) (Jombart et al., 2010) of the 7 clusters identified with Mclust and fitting a model built using 40 principal components (PCs) and 8 linear discriminants. The number of PCs to use to build the model was determined via cross-validation by building 1000 different models per PC with an 80-20 split of training/test data and selecting the combination that provided the maximum correct predictions with the lowest number of PCs. Further merging of the clusters was further

assessed using DAPC to find the differences between the three main populations of cells identified (A, B and Fulv7) and built with 40 PCs and 6 linear discriminants. Most important genes to discriminate the cell populations were determined using non-parametric Mann-Whitney test. False discovery rate was controlled via Benjamini and Hochberg method.

shRNA knockdown

The inducible Dharmacon TRIPZ lentiviral shRNA was used to stably down-regulate ALDH1A3 mRNA expression levels (ALDH1A3KD - Dharmacon, V3THS_378581; V3THS_378584; V3THS_378585).

Statistical analysis

P values less that 0.05 were considered significant (* $p<0.05$, ** $p<0.01$, *** $p<0.001$). Results are presented as the mean of at least 3 independent experiments ± Standard Error of the Mean (SEM) or Standard Deviation (SD).

SUPPLEMENTAL REFERENCES

Azur, M. J., Stuart, E. A., Frangakis, C., Leaf, P. J. (2011). Multiple Imputation by Chained Equations: What is it and how does it work? Int J Methods Psychiatr Res 20, 40-49.

Breitling, R., Armengaud, P., Amtmann, A., Herzyk, P. (2004). Rank products: a simple, yet powerful, new method to detect differentially regulated genes in replicated microarray experiments. FEBS Lett 573, 83-92.

Fluidigm Corporation (2014). Fluidigm® SINGuLAR™ Analysis Toolset 2.0 R package. v3.6.2

Fluidigm Corporation (2016). Doublet Rate and Detection on the C1 IFCs White Paper, PN 101-2711 A1.

Gautier, L., Cope, L., Bolstad, B. M., Irizarry, R. A. (2004). affy--analysis of Affymetrix GeneChip data at the probe level. Bioinformatics 20, 307-315.

Jombart, T., Devillard, S., Balloux, F. (2010). Discriminant analysis of principal components: a new method for the analysis of genetically structured populations. BMC Genet 11, 94.

Leek, J. T., Johnson, W. E., Parker, H. S., Fertig, E. J., Jaffe, A. E., Storey, J. D., Zhang, Y., Torres, L. C. (2019). sva: Surrogate Variable Analysis. R package version 3.34.0.

Shaw, F. L., Harrison, H., Spence, K., Ablett, M. P., Simões, B. M., Farnie, G., Clarke, R. B. (2012). A detailed mammosphere assay protocol for the quantification of breast stem cell activity. J Mammary Gland Biol Neoplasia 17, 111-117.

Scrucca, L., Fop, M., Murphy, T. B., Raftery, A. E. (2016). mclust 5: Clustering, Classification and Density Estimation Using Gaussian Finite Mixture Models. R J 8, 289-317.

Ward, J. H. (1963). Hierarchical Grouping to Optimize an Objective Function. Journal of the American Statistical Association 58, 236-244.
