# Supplementary material for: Increased Expression of Interleukin-1 Receptor Characterizes Anti-estrogen-Resistant ALDH+ Breast Cancer Stem Cells
Source: Stem Cell Reports. 2020 Jul 23;15(2):307–16. doi: 10.1016/j.stemcr.2020.06.020 (PMC7419713; doi:10.1016/j.stemcr.2020.06.020)
Supplement: Document S2. Article plus Supplemental Information [file mmc5.pdf]

Stem Cell Reports
Report
OPEN ACCESS

Increased Expression of Interleukin-1 Receptor Characterizes Anti-estrogen-Resistant ALDH$^+$ Breast Cancer Stem Cells

Aida Sarmiento-Castro,[1] Eva Caamaño-Gutiérrez,[2] Andrew H. Sims,[3] Nathan J. Hull,[1] Mark I. James,[1] Angélica Santiago-Gómez,[1] Rachel Eyre,[1] Christopher Clark,[1] Martha E. Brown,[4] Michael D. Brooks,[4] Max S. Wicha,[4] Sacha J. Howell,[1] Robert B. Clarke,[1,*] and Bruno M. Simões[1,*]
[1]Manchester Breast Centre, Division of Cancer Sciences, University of Manchester, Manchester M20 4GJ, UK
[2]Technology Directorate, Institute of Systems, Molecular & Integrative Biology, University of Liverpool, Liverpool L69 7ZB, UK
[3]Applied Bioinformatics of Cancer Group, University of Edinburgh Cancer Research UK Centre, Edinburgh EH4 2XR, UK
[4]Comprehensive Cancer Center, University of Michigan, Ann Arbor, MI 48109, USA
*Correspondence: robert.clarke@manchester.ac.uk (R.B.C.), bruno.simoes@manchester.ac.uk (B.M.S.)
https://doi.org/10.1016/j.stemcr.2020.06.020

SUMMARY

Estrogen-receptor-positive breast tumors are treated with anti-estrogen (AE) therapies but frequently develop resistance. Cancer stem cells (CSCs) with high aldehyde dehydrogenase activity (ALDH$^+$ cells) are enriched following AE treatment. Here, we show that the interleukin-1β (IL-1β) signaling pathway is activated in ALDH$^+$ cells, and data from single cells reveals that AE treatment selects for IL-1 receptor (IL1R1)-expressing ALDH$^+$ cells. Importantly, CSC activity is reduced by an IL1R1 inhibitor in AE-resistant models. Moreover, IL1R1 expression is increased in the tumors of patients treated with AE therapy and predicts treatment failure. Single-cell gene expression analysis revealed that at least two subpopulations exist within the ALDH$^+$ population, one proliferative and one quiescent. Following AE therapy the quiescent population is expanded, which suggests CSC dormancy as an adaptive strategy that facilitates treatment resistance. Targeting of ALDH$^+$IL1R1$^+$ cells merits testing as a strategy to combat AE resistance in patients with residual disease.

INTRODUCTION

Breast cancer (BC) represents 25% of all cancer diagnoses and is the fifth most common cause of death in women worldwide. Approximately 80% of BCs are positive for estrogen receptor expression (ER$^+$ tumors) and are treated with anti-estrogen (AE) adjuvant therapies such as tamoxifen or fulvestrant. Despite the clear benefit of these drugs at reducing tumor recurrence, *de novo* or acquired resistance often occurs (Pan et al., 2017).

Cancer stem cells (CSCs) are a cellular population endowed with self-renewal properties, which are responsible for tumor progression and metastasis (Reya et al., 2001). Aldehyde dehydrogenase (ALDH) activity is reported to be a CSC marker in human BC cells (Ginestier et al., 2007). ALDH$^+$ cells are ER-negative and likely to be resistant to the direct effects of AE therapy (Honeth et al., 2014). We have previously established that ALDH$^+$ cells drive therapeutic resistance in ER$^+$ BC tumors (Simões et al., 2015).

Intra-tumor heterogeneity within BCs hinders accurate diagnosis and effective treatment. Understanding of the cellular diversity within the CSC population, especially at the single-cell level, is limited. Given the importance of ALDH$^+$ cells in promoting AE resistance, we investigated the gene expression pattern of this cellular population at the single-cell level. This study reveals a previously uncharacterized level of heterogeneity within AE-resistant CSCs and identifies IL1R1 as a potential target in refractory and dormant BCs.

RESULTS

ALDH$^+$ Cells from AE-Treated ER$^+$ BCs Have Greater Breast CSC Activity Than ALDH$^-$ Cells

Previous research reported by our group (Simões et al., 2015) established that AE treatment of BC patient-derived xenograft tumors in mice enriches for breast CSCs (BCSCs) with high ALDH enzymatic activity. To further investigate this AE-resistant population, we isolated ALDH$^+$ and ALDH$^-$ cells from eight metastatic ER$^+$ BCs undergoing AE therapies. There was significant inter-individual variation in the percentage of ALDH$^+$ cells (range 0.32%–27.3%) (Figures 1A and S1A). Importantly, ALDH$^+$ cells exhibited significantly greater BCSC activity as assessed by mammosphere formation than ALDH$^-$ cells in seven out of eight patient samples, and in four of these samples the mammosphere-forming efficiency (MFE) was increased by more than 3-fold (Figure 1B). On average, ALDH$^+$ cells from the eight metastatic BC samples showed 3.8-fold greater MFE than ALDH$^-$ cells ($p = 0.001$) (Figure 1C). Next, we investigated the *in vivo* tumor-initiating capabilities of ALDH$^+$ cells isolated from the ER$^+$ cell line MCF-7 following 6-day *in vitro* treatment with the AEs tamoxifen or fulvestrant (Figure 1D). Injection of 1,000 ALDH$^+$ cells consistently gave rise to bigger tumors compared with the same number of ALDH$^-$ cells, significantly so in tamoxifen- and fulvestrant-treated cells (Figure 1E). Extreme limiting dilution analysis revealed that on average the number of tumor-initiating cells was 4.2-fold higher in

This is an open access article under the CC BY license (http://creativecommons.org/licenses/by/4.0/).

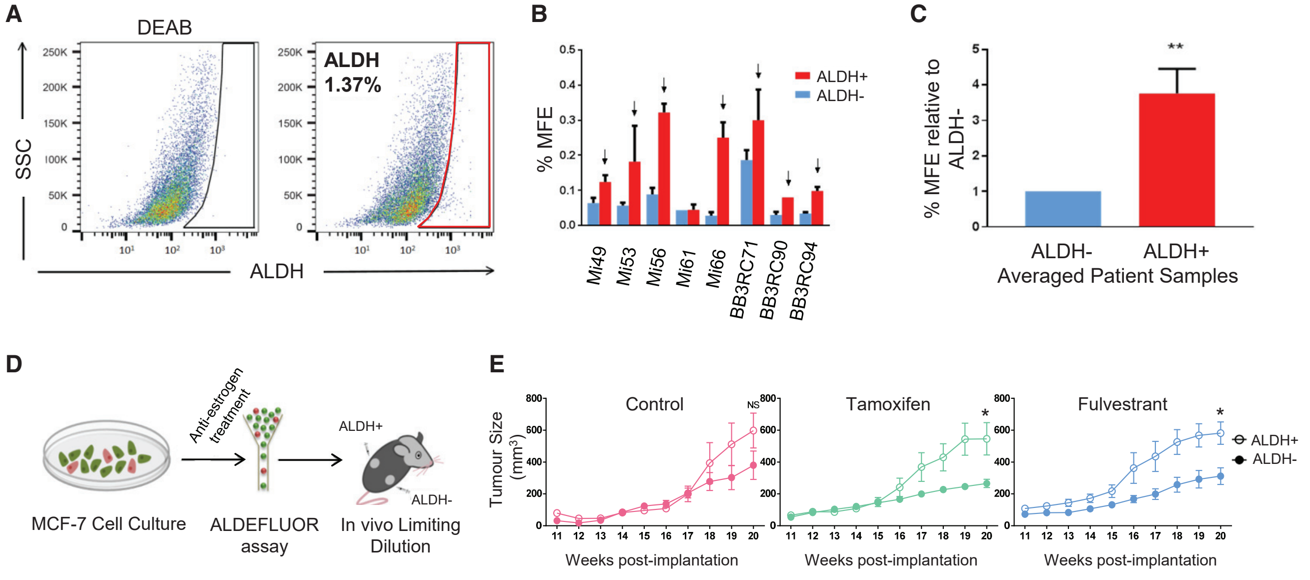

F

		MCF-7 treatment groups					
		Control		Tamoxifen		Fulvestrant	
		ALDH-	ALDH+	ALDH-	ALDH+	ALDH-	ALDH+
Cell Number	10,000	2/4	3/4	2/4	3/4	0/3	2/3
	1,000	1/3	3/3	1/4	2/4	2/4	4/4
	100	0/4	1/4	0/4	1/4	0/4	0/4
Tumour Initiating Cell Frequency (95% CI)		1:1,044	**1:274**	1:1,069	**1:365**	1:1,670	**1:291**
P value compared to ALDH- (X^2)		N/A	**0.05**	N/A	0.14	N/A	**0.02**

Figure 1. AE-Treated ALDH$^+$ Cells from ER$^+$ BC Cells Have Greater BCSC Activity Than ALDH$^-$ Cells *In Vitro* and *In Vivo*

(A) Representative fluorescence activated cell sorting (FACS) plot showing the ALDH$^+$ population identified through the Aldefluor assay for an individual patient sample. ALDH$^+$ cells (red gate) were discriminated from ALDH$^-$ cells using the diethylaminobenzaldehyde (DEAB) control.

(B) Bar chart shows mammosphere-forming efficiency (MFE) percentage of ALDH$^+$ cells (red) and ALDH$^-$ cells (blue) from ER$^+$ metastatic BCs undergoing AE therapies.

(C) Bar chart illustrates fold change in MFE percentage between ALDH$^+$ and ALDH$^-$ cells across eight different patient samples.

(D) Schematic overview of the *in vivo* transplantation assay to test tumor formation capacity between ALDH$^+$ and ALDH$^-$ MCF-7 cells. MCF-7 cells were pre-treated *in vitro* for 6 days with control (ethanol), tamoxifen (1 μM) or fulvestrant (0.1 μM) followed by the Aldefluor assay. ALDH$^+$ and ALDH$^-$ cells were FACS sorted, counted using trypan blue, and engrafted into the left and right flank, respectively, of the same NSG mice.

(E) Averaged tumor growth from control (pink; left panel), tamoxifen (green; middle panel), or fulvestrant-treated (blue; right panel) cells. 1,000 ALDH$^+$ (hollow circles) and 1,000 ALDH$^-$ (filled circles) cells are represented. $*p \leq 0.05$ (two-tail, two-sample equal-variance t test). Number of mice per condition = 4 (vehicle-treated mice, n = 3). Data shown as mean ± SEM.

(F) Table shows extreme limiting dilution analysis from *in vivo* injections of ALDH$^+$ and ALDH$^-$ cells (10,000; 1,000; 100 cells) to assess tumor-initiating cell frequency. Tumor growth was assessed at week 20 and is represented as mice positive for growth/mice tested for each cell number.

See also Figure S1.

ALDH$^+$ compared with the non-BCSC ALDH$^-$ cells in all three conditions tested (Figure 1F). As few as 100 ALDH$^+$ cells gave rise to tumors in mice whereas 100 ALDH$^-$ cells failed to do so. These results highlight the increased tumor-initiating capabilities of the ALDH$^+$ population in comparison with ALDH$^-$ cells, implying the need to characterize this population of CSCs that survive AE therapies.

Transcriptomic Characterization of ALDH$^+$ Cells in Therapy-Resistant Patient Samples

To better understand the development of resistance to AE therapies in ER$^+$ BC patients, we interrogated and compared the gene expression pattern between ALDH$^+$ and ALDH$^-$ cells in nine ER$^+$ metastatic samples (Figure 2A). All patients had progressive disease since they required

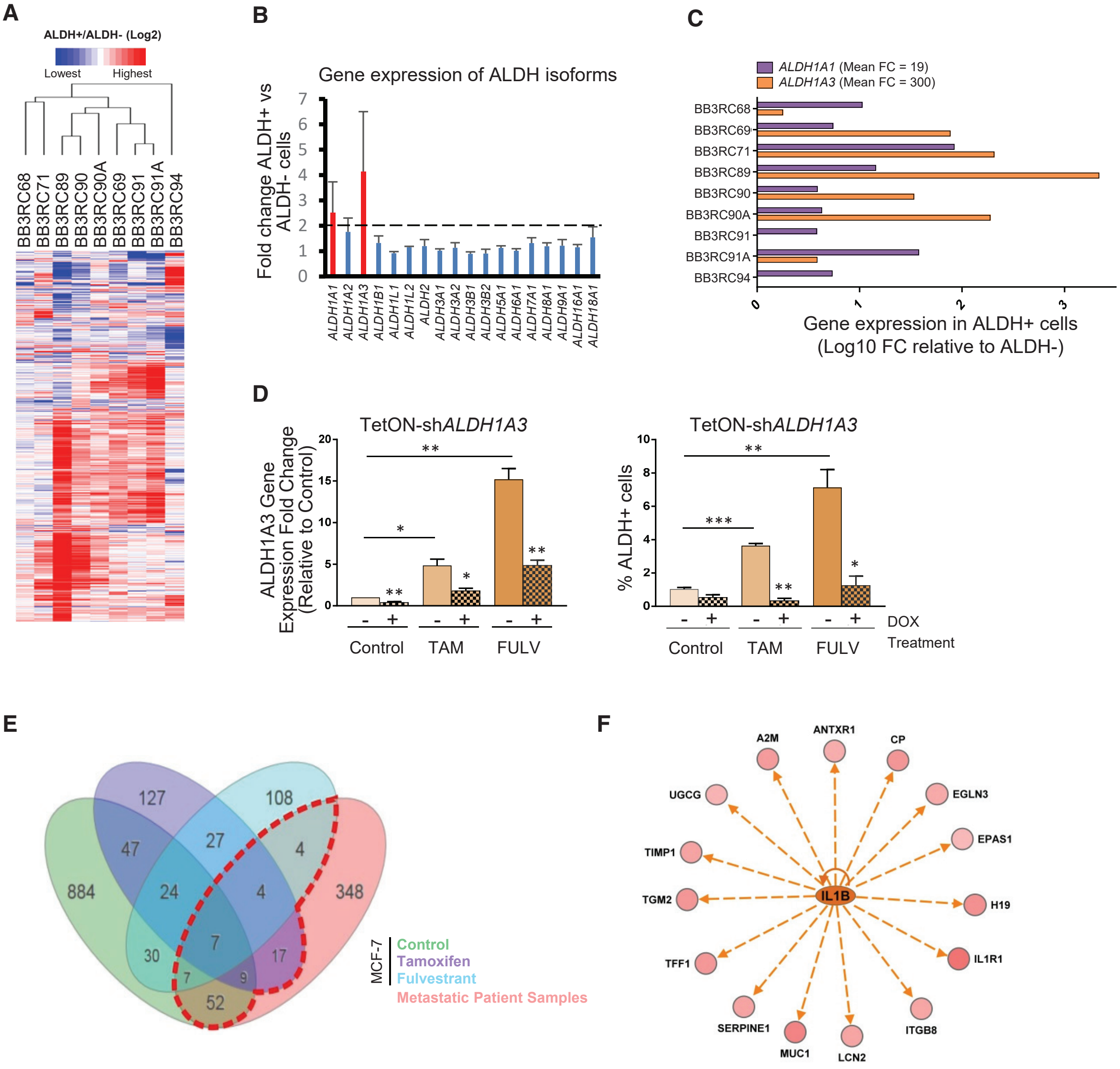

Figure 2. ALDH$^+$ Cells from ER$^+$ Metastatic Samples Show a Distinct Gene Expression Pattern Compared with ALDH$^-$ Cells

(A) Heatmap illustrating the 599 differentially expressed genes (447 up, 152 down) between ALDH$^+$ and ALDH$^-$ cells (red color shows gene upregulation, green shows downregulation in ALDH$^+$ relative to ALDH$^-$ cells identified by pairwise rank products with a threshold probability of false positives <0.05) from metastatic ER$^+$ patient BCs.

(B) Gene expression fold change (FC) between ALDH$^+$ and ALDH$^-$ cells of 18 ALDH isoforms detected in the Affymetrix array data. Mean FC for all metastatic samples (n = 9) is represented for each isoform. Red bar indicates isoforms with FC higher than 2.

(C) qPCR analysis of *ALDH1A1* and *ALDH1A3* gene expression in the nine patient metastatic samples that were used in the Affymetrix array. Data are shown as log_{10} FC between ALDH$^+$ and ALDH$^-$ cells. Mean linear FC of the two ALDH isoforms for all samples is shown.

(D) A stably transduced inducible sh*ALDH1A3* MCF-7 cell line was treated with control, tamoxifen (TAM), or fulvestrant (FULV) for 6 days concomitantly with (filled pattern) or without (solid bars) doxycycline (DOX). *ALDH1A3* mRNA levels were examined by qPCR (left) and percentage of ALDH$^+$ cells was assessed using the Aldefluor assay (right). Data of at least three independent experiments are shown (*p < 0.05, **p < 0.01, ***p < 0.001).

(E) Venn diagram illustrates meta-analysis of the MCF-7 cell line (control, tamoxifen, fulvestrant-treated ALDH$^+$ versus ALDH$^-$ cells) and the patient Affymetrix data (ALDH$^+$ versus ALDH$^-$ cells). iPathway guide software tool (AdvaitaBio) was used to plot the diagrams. The red dashed-line box indicates the 100 genes that are commonly differentially expressed in ALDH$^+$ cells of patient samples and MCF-7 cell line. The log_2 FC cutoff applied to the ALDH$^+$ versus ALDH$^-$ cells obtained from the meta-analysis data was 0.6.

(legend continued on next page)

pleural effusion or ascitic drainage as palliative care, but while six samples were treated with endocrine therapies the other three were endocrine therapy-naive (Table S1). Overall, 599 genes were found to be differentially expressed ($p \leq 0.05$) between the two ALDH cell populations among the 18,752 genes with measured expression (Table S2).

To identify which isoforms of ALDH are responsible for the Aldefluor activity of $ALDH^+$ cells in metastatic ER^+ patient samples, we investigated the mRNA expression levels of the 18 detected ALDH isoforms in our patient sample dataset. *ALDH1A1* and *ALDH1A3* showed the greatest fold change (FC) between $ALDH^+$ and $ALDH^-$ cells with a mean FC higher than 2 (Figure 2B). Validation by qRT-PCR confirmed the elevated expression of *ALDH1A1* and *ALDH1A3* isoforms in the $ALDH^+$ compared with $ALDH^-$ population, with a considerably higher averaged linear FC of *ALDH1A3* (300-fold) than *ALDH1A1* (19-fold) across the nine patient samples (Figure 2C). Interestingly, we also found that 6 days of AE treatment significantly upregulated *ALDH1A3* mRNA levels in two ER^+ cell lines, MCF-7 and T47D (Figure S2A). Therefore, we used a doxycycline-inducible short-hairpin RNA system to test the effects of *ALDH1A3* silencing on AE resistance. *ALDH1A3* was stably downregulated by 58% compared with transfected cells not exposed to doxycycline, and there was a significant decrease in the induction of *ALDH1A3* mRNA levels following AE treatment in the knockdown (KD) cells (Figure 2D, left). The enrichment in the $ALDH^+$ cell population after tamoxifen and fulvestrant treatments was significantly reduced in the ALDH1A3 KD cells (Figure 2D, right), indicating the importance of the ALDH1A3 isoform in $ALDH^+$ cell population after AE therapy.

We also interrogated the gene expression profile of $ALDH^+$ and $ALDH^-$ populations from AE-treated MCF-7 cells. The meta-analysis from the patient and cell line microarray datasets ($FC \geq \pm 1.5$ and $p \leq 0.05$) revealed 100 genes commonly shared between $ALDH^+$ cells of patient samples and $ALDH^+$ cells of the MCF-7 cell line (Figure 2E and Table S3). Ingenuity Pathway Analysis for these genes predicted activation of eight upstream regulators (Z score ≥ 2.5), including several cytokines; for example, interleukin-1β (IL-1β) (Figure S2B). Of the 100 genes identified in the $ALDH^+$ cell population, 15 were predicted to be regulated by IL-1β and these were all upregulated in the $ALDH^+$ cells, which is consistent with activation of IL-1β signaling (Figure 2F). This activation was more obvious in the $ALDH^+$ cells of AE-resistant samples, since 14 out of the 15 IL-1β-regulated genes were expressed at lower levels in the $ALDH^+$ cells of the endocrine therapy-naive samples (Figure S2C). One of these genes was interleukin-1 receptor type 1 (IL1R1), which binds and transmits the signal of both IL-1α and IL-1β.

AE Treatment Selects for IL1R1-Expressing $ALDH^+$ Cells

To study the effects of AE treatment on the $ALDH^+$ population at the single-cell level, we analyzed the expression of *IL1R1* and *ALDH1A3* in 178 individual $ALDH^+$ cells following tamoxifen or fulvestrant treatment. Sorted $ALDH^+$ cells were injected and captured in the C1 system, followed by microscopic examination of cell singlets (Figure S3A). When comparing *IL1R1* gene expression profiles between control and AE-treated $ALDH^+$ cells, we observed that gene expression levels of *IL1R1* increased significantly following tamoxifen and fulvestrant treatment (Figures 3A and 3B). In contrast, *ALDH1A3* expression was high in nearly all $ALDH^+$ cells, with or without therapy, as expected (Figures 3A and 3B). *IL1R1* gene expression density plots revealed that control $ALDH^+$ cells match a bimodal distribution with two distinct transcriptomic states: a population that comprises the majority of cells, which show none or very low *IL1R1* gene expression levels, and a small population of cells showing high *IL1R1* levels. However, following AE therapy the vast majority of $ALDH^+$ cells show high *IL1R1* gene expression levels (Figures 3B and S3B). These results reveal the existence of cellular diversity within the $ALDH^+$ population that can be unraveled by single-cell gene expression profiling, and highlight *IL1R1* as an important gene in AE-resistant BCSCs. Indeed, MCF-7 tamoxifen- and fulvestrant-resistant cell sublines express significantly higher levels of both *ALDH1A3* and *IL1R1* genes when compared with parental cell sublines (Figure 3C; Coser et al., 2009). In addition, AE-resistant MCF-7 cell lines show increased MFE when compared with the parental AE-sensitive cell line, which can be significantly reduced by anakinra, a recombinant form of human IL1R1 antagonist (Figure 3D).

To determine the clinical significance of identifying IL1R1 as facilitating AE resistance, we assessed *IL1R1* gene expression levels in patient breast tumors. Consistent with our cell line data, we found *IL1R1* gene expression levels to be increased in breast tumors following short-term administration of fulvestrant to patients (Figure 3E; Patani et al., 2014). In addition, we observed that *IL1R1* expression was significantly increased upon short- and long-term aromatase inhibitor (AI) treatment compared

(F) Ingenuity Pathway Analysis diagram showing that IL-1β signaling is predicted to be activated (orange color) in the $ALDH^+$ cell population. Straight arrows indicate network of 15 genes, predicted to be regulated by IL-1β, that were upregulated (in red) in $ALDH^+$ cells.
See also Figure S2.

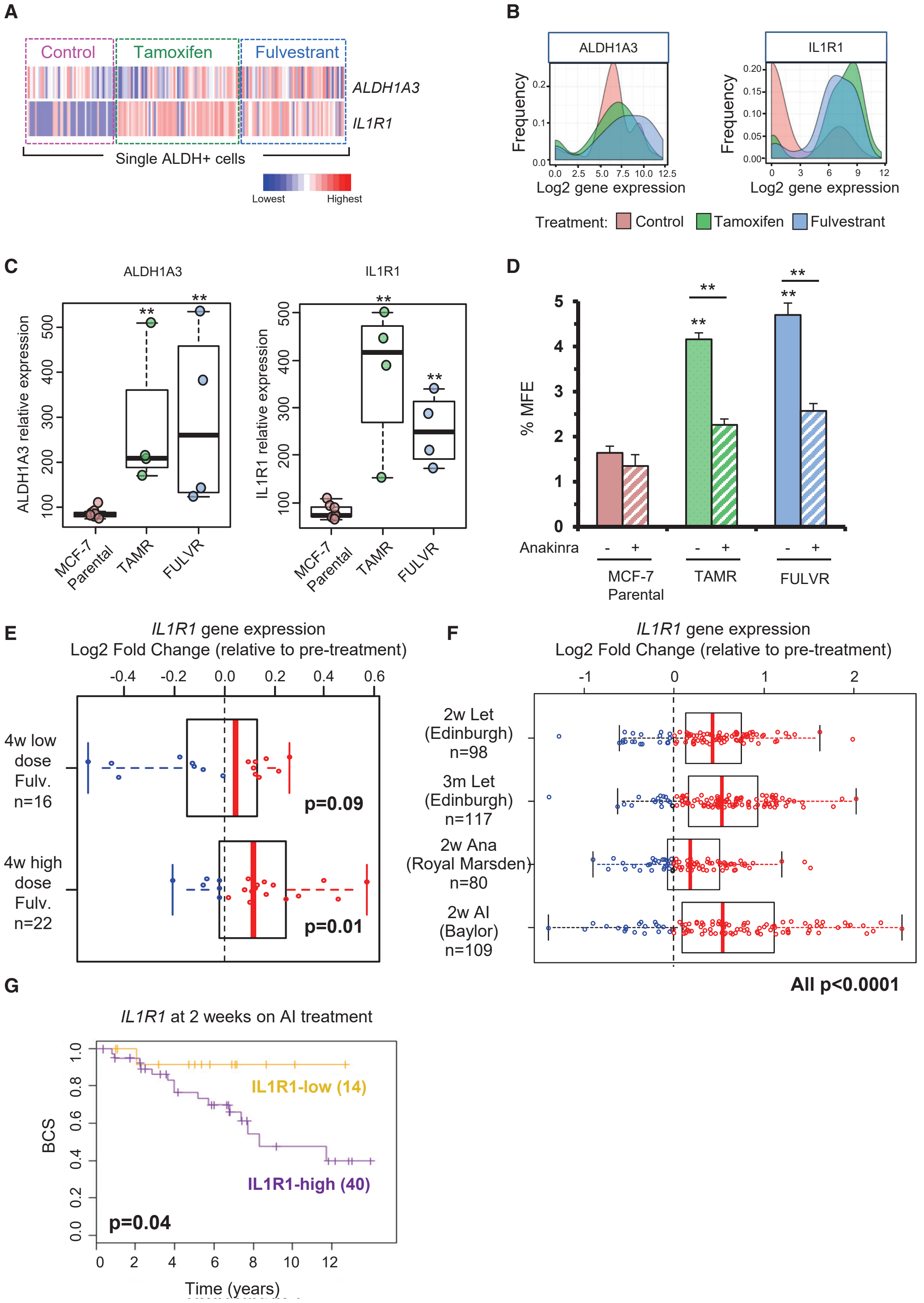

(legend on next page)

with baseline levels in four different patient cohorts totaling 404 patients (Dunbier et al., 2013; Ellis et al., 2017; Turnbull et al., 2015) (Figure 3F). Notably, we also found that elevated expression of *IL1R1* in ER+ patients who had been treated with AI for 2 weeks was significantly associated with a poor outcome (Figure 3G).

Single-Cell RNA Profiling Identifies a Dormant ALDH+ Population that Is Expanded after AE Treatment

IL1R1 and *ALDH1A3* single-cell gene expression revealed heterogeneity within ALDH+ cells; therefore, we decided to investigate the existence of putative subpopulations within the ALDH+ cell population. We examined the mRNA expression level of 68 genes across 377 single ALDH+ MCF-7 cells following control, tamoxifen, or fulvestrant treatment. The 68-gene list (see Supplemental Experimental Procedures) comprised key regulators associated with stemness, self-renewal pathways, and markers related to ALDH+ cells that were identified in the whole gene expression dataset (Figure 2). A Gaussian mixture model approach to estimate and assign clusters to the cells predicted the existence of seven different cellular ALDH+ populations (control: 1 and 2, tamoxifen: 3 and 4, fulvestrant: 5, 6, and 7) (Figure 4A). Some of these initial clusters were merged, based on their gene expression similarities using Ward's hierarchical clustering on Euclidean distance coupled with bootstrapping to estimate branch robustness. This analysis resulted in two major populations of cells, population A and population B, which were both made of clusters from the three different treatments, and a small population of fulvestrant-treated cells (fulvestrant 7) that were distinct from the rest of the cells (Figure 4B). Next, we applied discriminant analysis of principal components (DAPC) to create a graphical representation of these three distinct populations (Figure 4C). The eight genes most associated with the first linear discriminant had the highest contribution to the separation of population B from the other populations (Figure 4D). Genes associated with cell proliferation, for example the cycle regulator *CCND1* and protein kinase *AKT1*, were downregulated in population B compared with population A, whereas the expression of the mesenchymal marker *SNAI2* was higher in the former (Figure 4E). Moreover, population A, which comprised the vast majority of the ALDH+ cells analyzed (82%), exhibited higher expression of proliferative markers *PCNA* and *KI67* in comparison with population B (Figure S4A). Interestingly, only 10% of the non-treated ALDH+ cells belonged to the quiescent population B; however, following tamoxifen and fulvestrant treatment the percentage of quiescent cells represented 44% and 19% of the total cells, respectively (Figure 4F). A recent study (Selli et al., 2019) investigated gene expression changes of dormant and acquired resistant ER+ tumors treated with an AI for more than 4 months. Notably, *ALDH1A1* and *ALDH1A3* gene expression levels were significantly increased in dormant tumors compared with acquired resistant tumors, which supports the existence of an ALDH+ dormant population after AE treatment (Figure 4G). Relative to pre-treatment, the dormant tumors also had significantly increased expression of both *ALDH1A1* and *ALDH1A3* as well as *IL1R1* and *SNAI2*, along with reduced *CCND1* (Figure S4B), consistent with the results above for the dormant

Figure 3. Single ALDH+ Cell Gene Expression in the MCF-7 Cell Line Identifies *IL1R1* Overexpression Following AE Treatment
(A) Heatmap of the relative expression across single ALDH+ cells (columns) for *ALDH1A3* and *IL1R1* genes (rows). Cells are ordered by treatment, i.e., control (left), tamoxifen (middle), and fulvestrant (right). Colors represent expression levels from highest (red) to lowest (blue).
(B) Density plots of gene expression in all single ALDH+ cells analyzed from the two different AE treatments and control.
(C) Box plots and scatterplots show ALDH1A3 and IL1R1 relative gene expression from MCF-7 parental/unselected clonal sublines (n = 7) compared with tamoxifen-resistant (TAMR, n = 4) and fulvestrant-resistant (FULVR, n = 4) clonal sublines (GEO: GSE14986 dataset). Data from sublines grown without drugs (MCF-7 parental and TAMR) or with fulvestrant (FULVR). p value for at least four biological replicates calculated with Wilcoxon test, $^{**}p < 0.01$.
(D) MCF-7 parental, tamoxifen-resistant (TAMR), and fulvestrant-resistant (FULVR) cells were pre-treated in adherence with 10 μg/mL anakinra or vehicle control in the presence of 10 ng/mL IL-1β for 72 h. MFE was assessed after pre-treatments. Data are presented as mean ± SEM of three experiments with at least three technical replicates each. $^{**}p < 0.01$.
(E) Box plot and scatterplot show *IL1R1* expression from ER+ BC after pre-surgical 4-week treatment with fulvestrant (low-dose, 250 mg or high-dose, 500 mg) compared with *IL1R1* expression before treatment (Patani et al., 2014). Data are presented as $\log_2$ FC. Each patient sample is displayed as a blue (downregulation) or red (upregulation) circle. p value calculated with paired Wilcoxon test.
(F) Box plot and scatterplot show *IL1R1* $\log_2$ FC gene expression in three different patient cohorts in response to 2 weeks (2w) or 3 months (3m) of letrozole (Let, Edinburgh dataset), anastrozole (Ana, Royal Marsden dataset), and AI (Baylor dataset) treatment compared with pre-treatment levels. Each patient sample is displayed as a blue (downregulation) or red (upregulation) circle. p value calculated with paired Wilcoxon test.
(G) Kaplan-Meier curves represent BC specific-survival (BCS) for *IL1R1*-high and *IL1R1*-low of a cohort of 54 ER+ BC patients (Edinburgh) who received 2 weeks of AI treatment. p value is based on a log-rank test.
See also Figure S3.

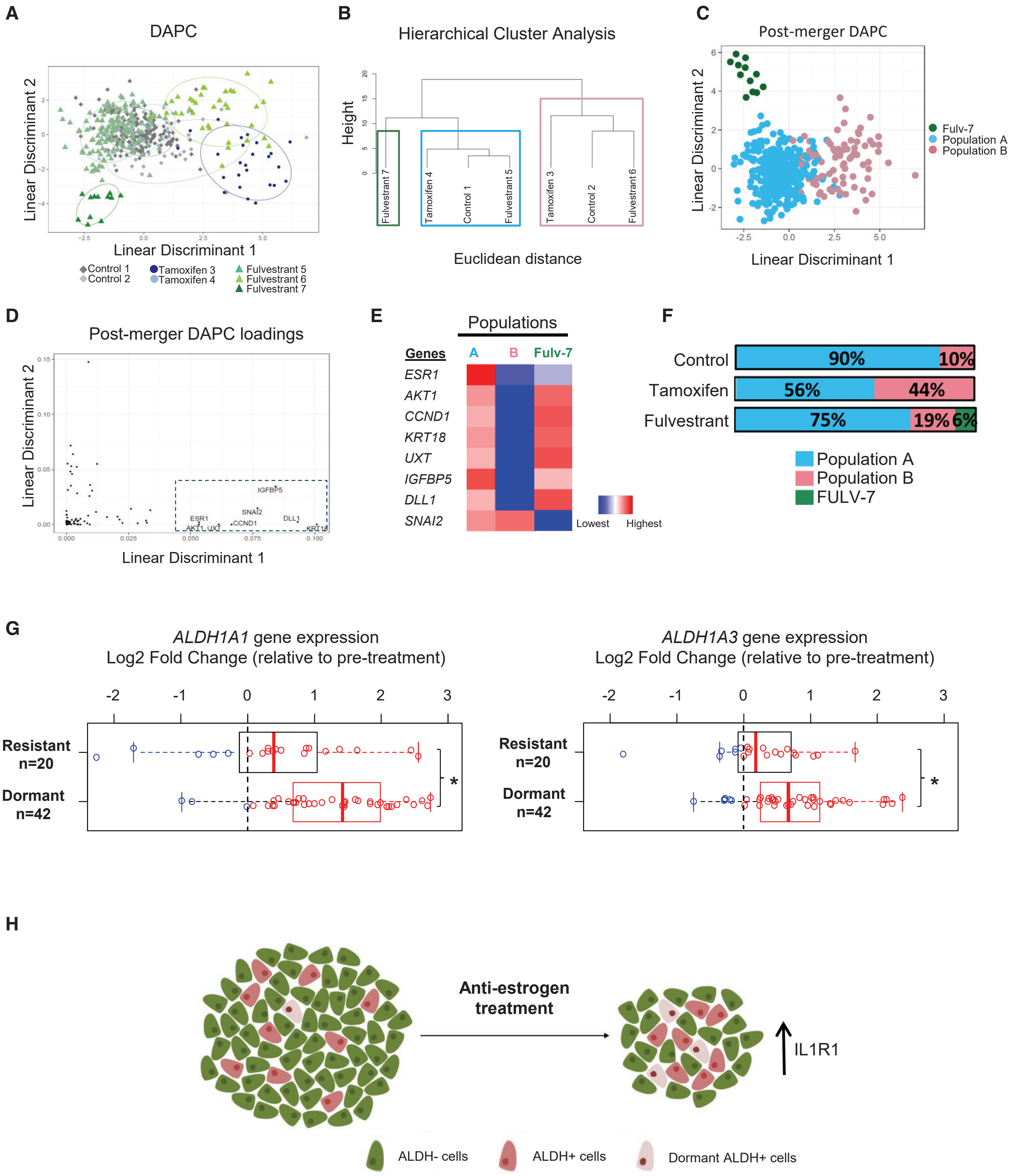

Figure 4. Single-Cell Gene Expression Data Reveal a Dormant ALDH$^+$ Population

(A) Scatterplot of the two first linear discriminants from discriminant analysis of DAPC analysis for 377 single ALDH$^+$ MCF-7 cells using as classifier the clusters identified through Mclust. The scatterplot shows the cluster of individual ALDH$^+$ cells (rhomboids: control group; circle: tamoxifen group; triangles: fulvestrant group). Control-treated ALDH$^+$ cells (gray) clustered within two groups (clusters 1 and 2),

(legend continued on next page)

population identified by single-cell analysis. These data suggest that AE resistance can be driven by non-proliferative dormant ALDH$^+$ cells and support a potential role for IL1R1-targeted therapy to overcome resistance in ER$^+$ BCs (Figure 4H).

DISCUSSION

Previously, we reported that ALDH$^+$ cells are resistant to AE therapy and that high ALDH1 expression predicts resistance in women treated with tamoxifen (Simões et al., 2015). Our findings here establish a role for the IL1R1 signaling pathway in the regulation of AE-resistant ALDH$^+$ BCSCs. We also identify heterogeneity in the ALDH$^+$ cell population and an expansion of a quiescent ALDH$^+$ subpopulation after AE therapies.

Firstly, we showed that BC cells contain a population of ALDH$^+$ cells that survive AE treatments, which maintain higher mammosphere-forming and tumor-initiating cell frequency than ALDH$^-$ cells. We next wanted to further characterize these cells and the mechanisms that drive them. ALDH1A1 and ALDH1A3 isoforms are both reported to be predictive biomarkers of poor clinical outcome in BC (Liu et al., 2014; Marcato et al., 2011), and we found them to be the most highly increased among 18 ALDH isoforms detected in metastatic patient-derived ALDH$^+$ BC cells. ALDH1A3 KD confirmed that this isoform is crucial for enriching the ALDH$^+$ population following AE treatment. These data support the growing body of literature describing the involvement of ALDH1A3 in cancer stemness, tumor progression, and poor prognosis.

We found that ALDH$^+$ cells have a different gene expression profile compared with ALDH$^-$ cells in both ER$^+$ metastatic patient samples and MCF-7 cells. In particular, genes that predicted activation of pro-inflammatory cytokine IL-1β signaling, including *IL1R1*, were expressed at higher levels in ALDH$^+$ cells. Furthermore, these genes were expressed at even higher levels in ALDH$^+$ cells of AE-treated compared with AE-naive patient samples. Gene expression analysis of ALDH$^+$ cells from AE-sensitive primary BC samples would validate our findings further but was not possible in the present study. By using single-cell gene expression profiling in the ALDH$^+$ cell population, we confirmed *IL1R1* to be significantly upregulated in AE-treated ALDH$^+$ cells compared with control cells. Moreover, AE-resistant cell lines express higher levels of *IL1R1* and display enriched CSC activity that is mainly dependent on IL-1β signaling, since it is significantly reduced by IL1R1 inhibition. Importantly, we found that expression of *IL1R1* is induced in the tumors of patients treated with AE therapies and predicts treatment failure. These data indicate that IL-1β signaling is likely to be important for CSCs to drive AE resistance in BC. *IL1β* expression correlates with increased aggressiveness and enhanced metastatic potential of BC cells, suggesting IL-1β as a potential biomarker for predicting which patients are likely to be diagnosed with BC metastasis, specifically to bone (Tulotta et al., 2019). Indeed, our group has recently demonstrated the importance of IL-1β-IL1R signaling in regulating stem cell activity in BC metastasis to the bone (Eyre et al., 2019). We established that bone marrow-derived IL-1β stimulates breast CSC colonization in the bone by inducing intracellular nuclear factor κB and Wnt signaling in breast CSCs. These findings suggest that metastatic dissemination is selecting for IL1R$^+$ CSCs that colonize the IL-1β-producing bone marrow.

Single-cell targeted transcriptome analysis revealed the existence of distinct clusters within ALDH$^+$ cells and the

Tamoxifen-treated ALDH$^+$ cells (blue) also clustered within two groups (clusters 3 and 4), and fulvestrant-treated ALDH$^+$ cells (green) clustered within three groups (clusters 5, 6, and 7). Pooled data of three independent experiments are shown.

(B) Ward hierarchical clustering of cell clusters using Euclidean distance of all the genes. Boxes represent clusters with an unbiased p value of >0.90 indicating that these clusters are robust, thus identifying three groups of cells: two major ones, renamed as population A (blue box) and population B (pink box) and a smaller one corresponding to Fulvestrant-7 (green box).

(C) Scatterplot of the DAPC analysis for single ALDH$^+$ MCF-7 cells after treatment using as classifier the clusters identified in (B). Linear discriminant 1 accounts for most of the differences between population B and the other two.

(D) Distribution of the gene importance to build linear discriminants 1 and 2. Genes above threshold 0.05 of linear discriminant 1 are labeled.

(E) Heatmap of relative gene expression across the three ALDH$^+$ populations identified (A, B, fulvestrant 7 [fulv-7]) for the eight most important genes in the separation between population B and the others. Colors represent expression levels from highest (red) to lowest (blue).

(F) Bar charts show the percentage contribution of each ALDH$^+$ subpopulation within the ALDH$^+$ cells treated with control, tamoxifen, or fulvestrant.

(G) Box plots and scatterplots show *ALDH1A1* and *ALDH1A3* expression from ER$^+$ dormant and acquired resistant tumors after 4 months of neoadjuvant treatment with letrozole compared with expression before treatment (Selli et al., 2019). Data are presented as $\log_2$ FC. Each patient sample is displayed as a blue (downregulation) or red (upregulation) circle. p value calculated with paired Wilcoxon test.

(H) Diagram showing that AE therapies do not target ALDH$^+$ cells and enrich for a dormant IL1R1$^+$ALDH$^+$ cell population.

See also Figure S4.

expansion of a quiescent ALDH$^+$ population (population B) after AE therapies. Heterogeneity within BCSCs of MCF-7 cells has previously been described using different CSC functional assays, such as mammospheres, growth in hypoxia, and PKH26 retention, to isolate single cells for gene expression analysis (Akrap et al., 2016). Our data suggest that population B represents a small population of non-dividing quiescent ALDH$^+$ cells that survive AE treatments, which may enable them to survive for long periods of time and eventually lead to late recurrence in ER$^+$ BC patients. This idea is supported by data from AI-induced dormant tumors that express increased levels of *ALDH1A1* and *ALDH1A3* genes. Recently, single-cell RNA profiling of normal breast samples identified four cell clusters within the ALDH$^+$ cell population (Colacino et al., 2018). Interestingly, population B resembles cluster 3 identified in this publication, which was characterized by high expression of mesenchymal markers, including *SNAI2*, and low expression of proliferative genes, such as *KI67*, *PCNA*, and *CCND1*. Population B expresses low levels of *AKT1*, and AKT1^{low} cancer cells have been reported to be quiescent cells that survive chemotherapy in breast tumors (Kabraji et al., 2017).

Combination therapies targeting both bulk tumor cells and BCSCs should reduce the probability of tumor relapse; therefore, pharmacological inhibitors that target CSC pathways have been highly pursued and are being tested in patients (Brooks et al., 2015). In our model, both proliferative and dormant AE-resistant BCSCs express *IL1R1*. This suggests that anti-IL1R1 therapies, such as anakinra or canakinumab (human anti-IL-1β monoclonal antibody), could represent a new strategy to target AE-resistant CSCs.

In conclusion, the present work contributes to our understanding of the cellular heterogeneity present in the AE-resistant BCSC population. Our work suggests that CSC dormancy is an adaptive strategy to evade AE treatments and supports the targeting of ALDH$^+$IL1R1$^+$ cells to reverse AE resistance. This work highlights the advantages of single-cell transcriptomic analysis, rather than bulk tissue, to interrogate the cellular heterogeneity within the ALDH$^+$ CSC population. Further understanding of the dormant ALDH$^+$ population that survives AE therapies, particularly using clinical samples, will provide new insights for prevention and treatment of recurrences of ER$^+$ BC.

EXPERIMENTAL PROCEDURES

A comprehensive description of the methodology is included in Supplemental Information.

Breast Cancer Samples

Consented, de-identified pleural effusion or ascitic fluids were collected at the Christie NHS Foundation Trust (UK) or the University of Michigan (USA). The clinicopathological details of the samples are shown in Table S4.

ALDH$^{+/-}$ Cell Isolation

BC cells were stained using the Aldefluor assay (STEMCELL Technologies) following the manufacturer's protocol and isolated using the Influx cell sorter (BD Biosciences).

Single-Cell Capture and Transcriptomics Profiling

Single ALDH$^+$ MCF-7 cells were captured within the C1 system using the medium C1 Single-Cell Preamp Integrated Fluidic Circuit (IFC, 10–17 μm) chips (Fluidigm, 100-5480). Individual cells were visualized using the Leica Widefield Low Light microscope. Cell loading, lysis, reverse transcription, and cDNA pre-amplification were performed within the C1 system following the manufacturer's instructions. We undertook three independent experiments that resulted in the single-cell transcriptomics profiling of 377 single cells. Data were acquired using the 96.96 Dynamic Array IFC Biomark chips (Biomark HD Real-Time PCR System, Fluidigm) to interrogate the expression of 68 TaqMan assays in each cell. Data analyses included different quality control steps and two iterative runs of clustering to identify cell populations. Further details on experimental design and data processing are described in the Single-Cell Data Analysis section of Supplemental Experimental Procedures.

Data and Code Availability

The Affymetrix data have been deposited in NCBI's Gene Expression Omnibus repository under series accession number GEO: GSE136287.

SUPPLEMENTAL INFORMATION

Supplemental Information can be found online at https://doi.org/10.1016/j.stemcr.2020.06.020.

AUTHOR CONTRIBUTIONS

B.M.S. and R.B.C. conceptualized the study. A.S.-C., R.B.C., and B.M.S. designed and carried out the experiments, performed data interpretation, and wrote the manuscript. E.C.-G. carried out bioinformatics single-cell analysis and wrote parts of the manuscript. A.H.S. performed bioinformatics analysis on Affymetrix data and patient data. M.I.J. created the knockdown cell line. C.C. and M.D.B. helped carry out single-cell experiments. M.E.B. provided patient samples from the Michigan cohort. N.J.H. performed mammosphere assays with cell lines. A.S.-G., R.E., M.S.W., and S.J.H. advised on experimental design and revised the manuscript. All authors edited and approved the final version.

ACKNOWLEDGMENTS

We are grateful to Breast Cancer Now (MAN-Q2, United Kingdom), Breast Cancer Research Foundation (United States), National Cancer Institute (R35 CA197585, United States), and Fashion Footwear Association of New York/QVC Presents Shoes-on-Sale™ (United States) for funding this research. B.M.S., R.B.C., and S.J.H. are supported by the NIHR Manchester

Biomedical Research Centre (IS-BRC-1215-20007, United Kingdom). We would like to thank the Medical Research Council (United Kingdom) that funded A.S.-C. with a Doctoral Training Scholarship (MR/K501311/1). We are grateful to the European Association of Cancer Research (United Kingdom) and the Windgate Foundation (United States) for funding travel fellowships to A.S.-C. We thank Prof. Daniel Hayes (University of Michigan) for assisting in the procurement of patient samples. M.S.W. has financial holdings in Oncomed Pharmaceuticals (United States) and receives research support from MedImmune (United States).

Received: August 29, 2019
Revised: June 20, 2020
Accepted: June 22, 2020
Published: July 23, 2020

REFERENCES

Akrap, N., Andersson, D., Bom, E., Gregersson, P., Stahlberg, A., and Landberg, G. (2016). Identification of distinct breast cancer stem cell populations based on single-cell analyses of functionally enriched stem and progenitor pools. Stem Cell Reports *6*, 121–136.

Brooks, M.D., Burness, M.L., and Wicha, M.S. (2015). Therapeutic implications of cellular heterogeneity and plasticity in breast cancer. Cell Stem Cell *17*, 260–271.

Colacino, J.A., Azizi, E., Brooks, M.D., Harouaka, R., Fouladdel, S., McDermott, S.P., Lee, M., Hill, D., Madden, J., Boerner, J., et al. (2018). Heterogeneity of human breast stem and progenitor cells as revealed by transcriptional profiling. Stem Cell Reports *10*, 1596–1609.

Coser, K.R., Wittner, B.S., Rosenthal, N.F., Collins, S.C., Melas, A., Smith, S.L., Mahoney, C.J., Shioda, K., Isselbacher, K.J., Ramaswamy, S., and Shioda, T. (2009). Antiestrogen-resistant subclones of MCF-7 human breast cancer cells are derived from a common monoclonal drug-resistant progenitor. Proc. Natl. Acad. Sci. U S A *106*, 14536–14541.

Dunbier, A.K., Ghazoui, Z., Anderson, H., Salter, J., Nerurkar, A., Osin, P., A'Hern, R., Miller, W.R., Smith, I.E., and Dowsett, M. (2013). Molecular profiling of aromatase inhibitor-treated postmenopausal breast tumors identifies immune-related correlates of resistance. Clin. Cancer Res. *19*, 2775–2786.

Ellis, M.J., Suman, V.J., Hoog, J., Goncalves, R., Sanati, S., Creighton, C.J., DeSchryver, K., Crouch, E., Brink, A., Watson, M., et al. (2017). Ki67 proliferation index as a tool for chemotherapy decisions during and after neoadjuvant aromatase inhibitor treatment of breast cancer: results from the American College of Surgeons Oncology Group Z1031 Trial (Alliance). J. Clin. Oncol. *35*, 1061–1069.

Eyre, R., Alférez, D.G., Santiago-Gómez, A., Spence, K., McConnell, J.C., Hart, C., Simões, B.M., Lefley, D., Tulotta, C., Storer, J., et al. (2019). Microenvironmental IL1β promotes breast cancer metastatic colonisation in the bone via activation of Wnt signalling. Nat. Commun. *10*, 5016.

Ginestier, C., Hur, M.H., Charafe-Jauffret, E., Monville, F., Dutcher, J., Brown, M., Jacquemier, J., Viens, P., Kleer, C.G., Liu, S., et al. (2007). ALDH1 is a marker of normal and malignant human mammary stem cells and a predictor of poor clinical outcome. Cell Stem Cell *1*, 555–567.

Honeth, G., Lombardi, S., Ginestier, C., Hur, M., Marlow, R., Buchupalli, B., Shinomiya, I., Gazinska, P., Bombelli, S., Ramalingam, V., et al. (2014). Aldehyde dehydrogenase and estrogen receptor define a hierarchy of cellular differentiation in the normal human mammary epithelium. Breast Cancer Res. *16*, R52.

Kabraji, S., Solé, X., Huang, Y., Bango, C., Bowden, M., Bardia, A., Sgroi, D., Loda, M., and Ramaswamy, S. (2017). AKT1low quiescent cancer cells persist after neoadjuvant chemotherapy in triple negative breast cancer. Breast Cancer Res. *19*, 88.

Liu, Y., Lv, D., Duan, J., Xu, S., Zhang, J., Yang, X., Zhang, X., Cui, Y., Bian, X., and Yu, S. (2014). ALDH1A1 expression correlates with clinicopathologic features and poor prognosis of breast cancer patients: a systematic review and meta-analysis. BMC Cancer *14*, 444.

Marcato, P., Dean, C.A., Pan, D., Araslanova, R., Gillis, M., Joshi, M., Helyer, L., Pan, L., Leidal, A., Gujar, S., et al. (2011). Aldehyde dehydrogenase activity of breast cancer stem cells is primarily due to isoform ALDH1A3 and its expression is predictive of metastasis. Stem Cells *29*, 32–45.

Pan, H., Gray, R., Braybrooke, J., Davies, C., Taylor, C., McGale, P., Peto, R., Pritchard, K.I., Bergh, J., Dowsett, M., and Hayes, D.F. (2017). 20-year risks of breast-cancer recurrence after stopping endocrine therapy at 5 years. N. Engl. J. Med. *377*, 1836–1846.

Patani, N., Dunbier, A.K., Anderson, H., Ghazoui, Z., Ribas, R., Anderson, E., Gao, Q., A'hern, R., Mackay, A., Lindemann, J., et al. (2014). Differences in the transcriptional response to fulvestrant and estrogen deprivation in ER-positive breast cancer. Clin. Cancer Res. *20*, 3962–3973.

Reya, T., Morrison, S.J., Clarke, M.F., and Weissman, I.L. (2001). Stem cells, cancer, and cancer stem cells. Nature *414*, 105–111.

Selli, C., Turnbull, A.K., Pearce, D.A., Li, A., Fernando, A., Wills, J., Renshaw, L., Thomas, J.S., Dixon, J.M., and Sims, A.H. (2019). Molecular changes during extended neoadjuvant letrozole treatment of breast cancer: distinguishing acquired resistance from dormant tumours. Breast Cancer Res. *21*, 2.

Simões, B.M., O'Brien, C.S., Eyre, R., Silva, A., Yu, L., Sarmiento-Castro, A., Alferez, D.G., Spence, K., Santiago-Gomez, A., Chemi, F., et al. (2015). Anti-estrogen resistance in human breast tumors is driven by JAG1-NOTCH4-dependent cancer stem cell activity. Cell Rep. *12*, 1968–1977.

Tulotta, C., Lefley, D.V., Freeman, K., Gregory, W.M., Hanby, A.M., Heath, P.R., Nutter, F., Wilkinson, J.M., Spicer-Hadlington, A.R., Liu, X., et al. (2019). Endogenous production of IL1B by breast cancer cells drives metastasis and colonization of the bone microenvironment. Clin. Cancer Res. *25*, 2769–2782.

Turnbull, A.K., Arthur, L.M., Renshaw, L., Larionov, A.A., Kay, C., Dunbier, A.K., Thomas, J.S., Dowsett, M., Sims, A.H., and Dixon, J.M. (2015). Accurate prediction and validation of response to endocrine therapy in breast cancer. J. Clin. Oncol. *33*, 2270–2278.

Stem Cell Reports, Volume *15*

Supplemental Information

Increased Expression of Interleukin-1 Receptor Characterizes Anti-estrogen-Resistant ALDH+ Breast Cancer Stem Cells

Aida Sarmiento-Castro, Eva Caamaño-Gutiérrez, Andrew H. Sims, Nathan J. Hull, Mark I. James, Angélica Santiago-Gómez, Rachel Eyre, Christopher Clark, Martha E. Brown, Michael D. Brooks, Max S. Wicha, Sacha J. Howell, Robert B. Clarke, and Bruno M. Simões

Supplementary Figure 1, Related to Figure 1

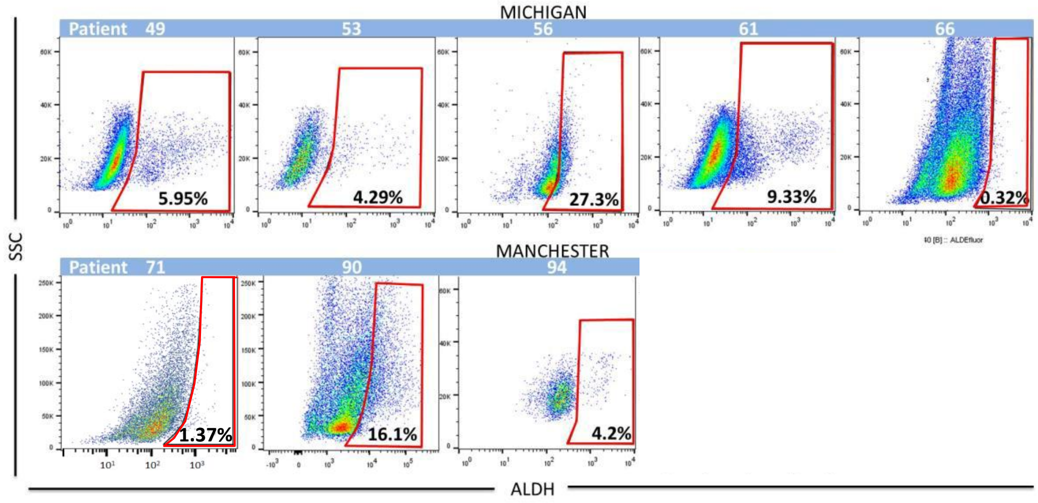

Figure S1. FACS plots showing percentage of ALDH+ cells, measured by the Aldefluor assay, in metastatic patient samples. ALDH+ cells (red box) from Michigan's biobank (top) and Manchester's biobank (bottom) patient-derived samples are shown.

Supplementary Figure 2, Related to Figure 2

A

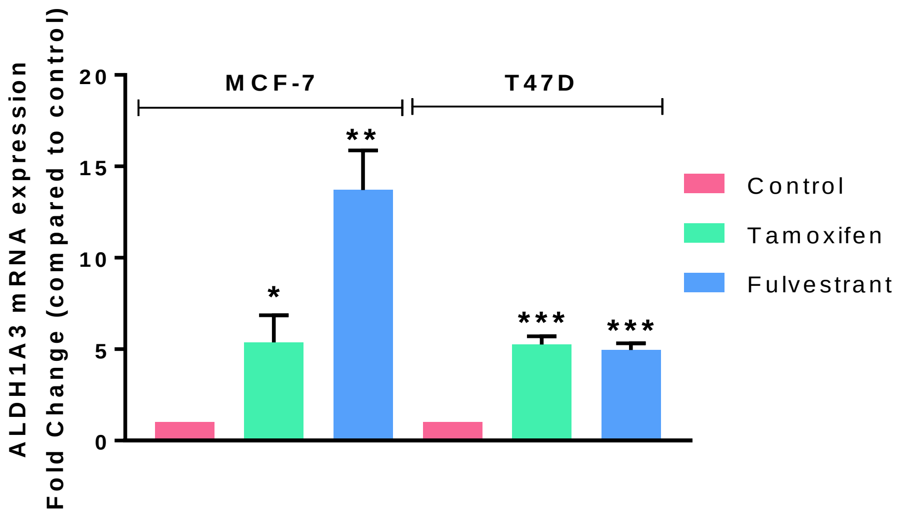

B

Upstream Regulator	Predicted Activation State	Activation z-score	p-value of overlap
TGFB1	Activated	3.29	0.00000468
AGT	Activated	3.04	0.0000123
SMARCA4	Activated	2.967	2.9E-09
IL6	Activated	2.925	0.00000036
TNF	Activated	2.875	7.56E-13
IL1B	Activated	2.674	0.000000434
OSM	Activated	2.545	0.0000608
ERK	Activated	2.53	4.18E-08

C

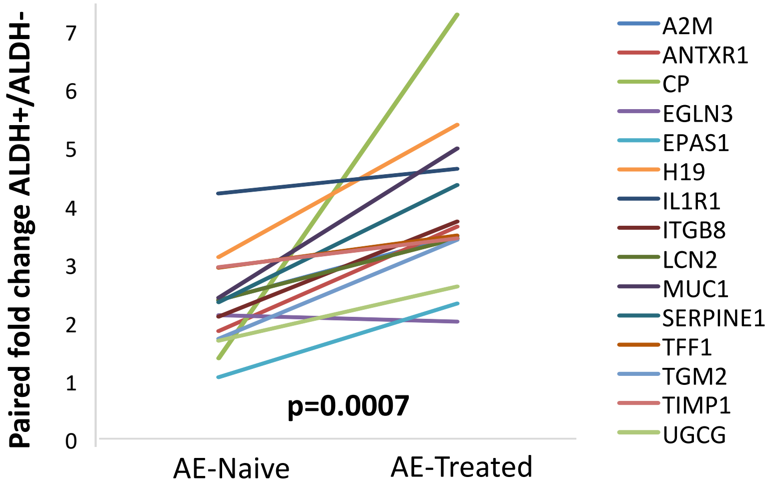

Figure S2. A) ALDH1A3 mRNA expression in MCF-7 and T47D cells following tamoxifen (green) and fulvestrant (blue) treatment compared to control (pink). Data of at least 3 independent experiments are shown (*p<0.05, **p<0.01, ***p<0.001). **B)** List of upstream regulators and respective predicted activation (with z-score ≥2.5) identified by Ingenuity Pathway Analysis (IPA) of 100 genes commonly expressed in ALDH+ cells of patient samples and MCF-7 cells. **C)** Gene expression of the 15 genes that predict IL1β activation in the ALDH+ cells of the 6 AE-treated metastatic samples (BB3RC68, BB3RC69, BB3RC71, BB3RC89, BB3RC91, BB3RC91A) and in the ALDH+ cells of the 3 AE-naïve metastatic samples (BB3RC90, BB3RC90A, BB3RC94). Graph shows the average paired fold change in ALDH+ vs ALDH- in both groups. P-value calculated with paired t-test comparing AE-treated with AE-naïve samples.

Supplementary Figure 3, Related to Figure 3

A

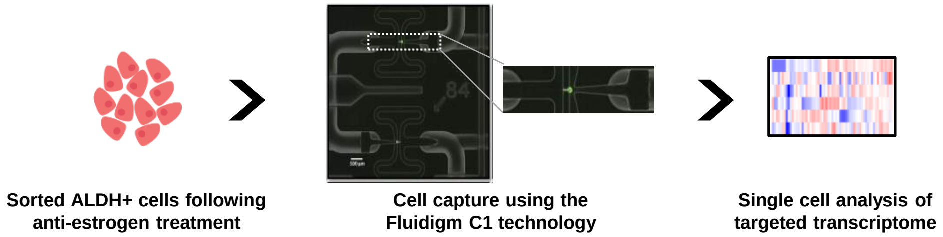

B

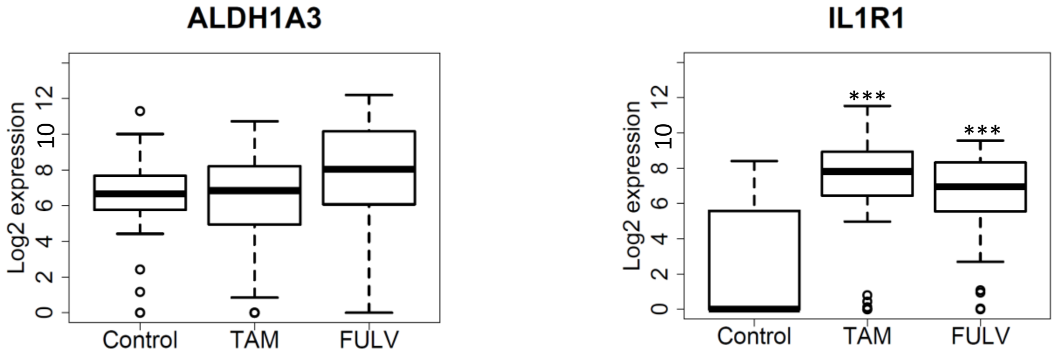

Figure S3. A) Schematic overview of the experimental approach to profile single ALDH+ cells. MCF-7 cells treated with either tamoxifen, fulvestrant or control were sorted into single cells and transcription profiles of genes of interest were obtained and analysed as described in the methods. **B)** Boxplots of *ALDH1A3* and *IL1R1* gene expression in cells treated with vehicle (Control), tamoxifen (TAM) and fulvestrant (FULV). Log2 expression distribution in all the cells is shown as boxes containing the interquartile ratio (first and third quartiles) with the median (bold line) and whiskers representing the 5–95% range. Kruskal-Wallis with Dunn's post-hoc correction was used to compare tamoxifen/fulvestrant treated cells versus control cells.***Pvalue<0.001

Supplementary Figure 4, Related to Figure 4

A

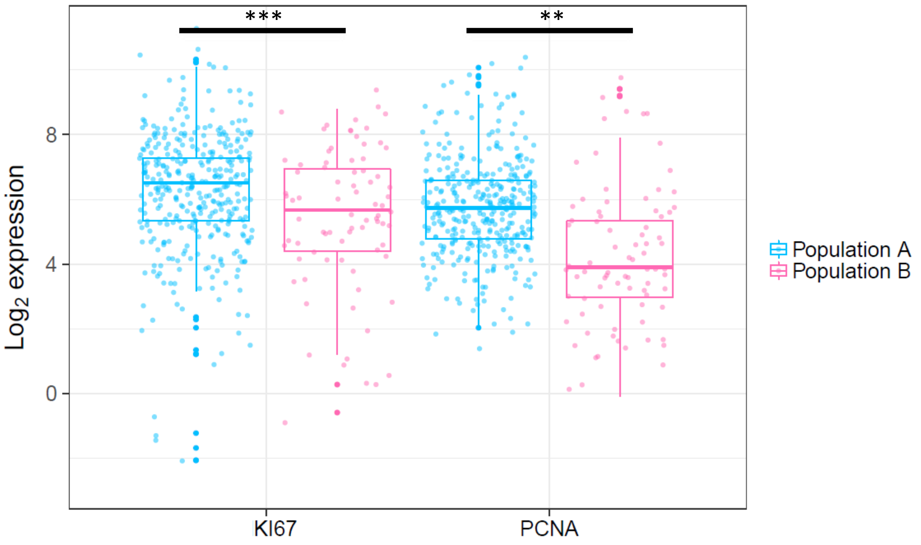

B

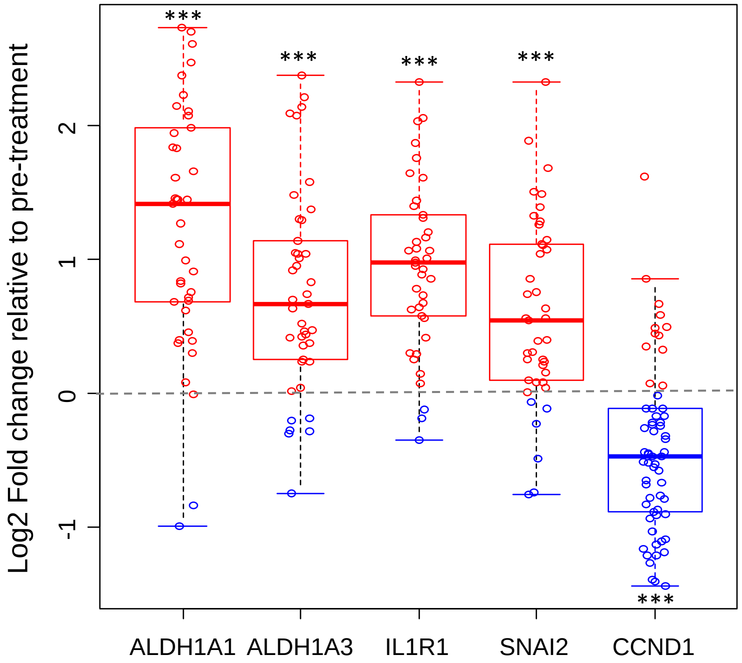

Figure S4. A) Boxplots of *Ki67* and *PCNA* gene expression in cells of Population A and B. Log2 expression distribution in all the cells is shown as boxes containing the interquartile ratio (first and third quartiles) with the median (bold line) and whiskers representing the 5–95% range. Each point within the plot represents a cell for the gene specified. Mann-Whitney U Test was used to compare Population B vs Population A. P values were adjusted for FDR with Benjamini and Hochberg method. (***Adj -Pvalue <0.001; **Adj-Pvalue<0.01). **B)** Boxplots show *ALDH1A1*, *ALDH1A3*, *IL1R1*, *SNAI2, CCND1* gene expression from ER+ dormant tumours after 4-months neoadjuvant treatment with letrozole compared to expression before treatment (Selli et al., 2019). Data is represented as Log2 fold change. Each patient sample is displayed as a blue (down-regulation) or red (up-regulation) circle. P-value calculated with paired Wilcoxon test.

Supplementary Tables

Table S1, Related to Figure 2. Timeline of anti-estrogen therapies received by each patient prior to sample collection.
Please refer to Supplemental spreadsheet file.

Table S2, Related to Figure 2. List of genes differentially expressed between ALDH+ and ALDH- cell populations in patient samples.
Please refer to Supplemental spreadsheet file.

Table S3, Related to Figure 2. List of differentially expressed genes commonly shared between ALDH+ cells of patient samples and ALDH+ cells of the MCF-7 cell line.
Please refer to Supplemental spreadsheet file.

Table S4, Related to Figure 1 and Figure 2. Clinico-pathological information of the Michigan and Manchester patient datasets. QS: Quick score.

Patient	ER	PR	Chemotherapy	Endocrine Therapy	Bone Therapy	Other Therapy	Metastasis
**Mi49**	+	+	Capecitabine<br>Cyclophosphamide<br>Doxorubicin<br>Eribulin<br>Etoposide<br>Gemcitabine<br>Ixabepilone<br>Taxol	Anastrozole<br>Fulvestrant<br>Goserelin	Zometa<br>Zolendronic acid	Anti-DLL4 antibody:<br>OMP-21M18	Bone<br>Brain<br>Pleura
**Mi53**	+	+	Cyclophosphamide<br>Doxorubicin<br>Taxol	Anastrozole<br>Tamoxifen	Zometa	-	Bone<br>Liver
**Mi56**	+	+	Capecitabine<br>Carboplatinum<br>Paclitaxel<br>Vinorelbine	Exemestane<br>Letrazole<br>Tamoxifen	Denosumab	-	Bone<br>Chest wall<br>Lymph node
**Mi61**	+	+	Cyclophosphamide<br>Docetaxel<br>Doxorubicin	Anastrozole<br>Exemestane	Denosumab	-	Bone<br>Pleura
**Mi66**	+	+	Capecitabine<br>Carboplatin<br>Gemcitabine	Arimidex<br>Tamoxifen	Denosumab	-	
**BB3RC68**	QS8		Capecitabine<br>Fluorouracil + Epirubicin + Cyclophosphamide (FEC)	Tamoxifen<br>Anastrazole<br>Fulvestrant	-	-	Bladder<br>Liver<br>Lung<br>Lymph nodes<br>Peritoneum
**BB3RC69**	96%	77%	-	Tamoxifen<br>Letrozole<br>Anastrazole	Pamidronate<br>Zoledronic Acid	-	Bone<br>Lymph node<br>Peritoneum
**BB3RC71**	54%	72%	Capecitabine<br>Eribulin<br>FEC<br>Taxol<br>Taxotere<br>Vinorelbine	Tamoxifen<br>Anastrazole<br>Fulvestrant<br>Exemestane	Pamidronate<br>Zoledronic Acid<br>Ibandronic Acid	Herceptin<br>Lapatinib	Bone<br>Liver<br>Pleura
**BB3RC89**	QS 8	QS 8	Capecitabine<br>FEC<br>Taxol	Tamoxifen<br>Letrozole<br>Exemestane	-	-	Bone<br>Liver<br>Lung
**BB3RC90-90A**	QS 8	QS 8	Capecitabine	-	-	-	Bone<br>Liver<br>Pleura
**BB3RC91-91A**	96%	98%	Docetaxel<br>FEC	Tamoxifen<br>Letrozole<br>Anastrazole	-	Everolimus	Bone<br>Liver<br>Omentum<br>Peritoneum
**BB3RC94**	+	+	Treatment naïve				Omentum<br>Peritoneum

N.B. Samples 90 and 90A are from the same patient but were taken at different time points. The same applies to samples 91 and 91A.

SUPPLEMENTAL EXPERIMENTAL PROCEDURES

Breast cancer samples

Metastatic fluids were collected at the Christie NHS Foundation Trust (UK) in accordance with local research ethics committee guidelines (study number: 05/Q1402/25) or the University of Michigan (study number: IRBMED 2001-0344/HUM00042204). Fluids were spun at 1000 g for 10 min at 4ºC and pellets were resuspended in Phosphate Buffered Saline (PBS). Erythrocytes and leucocytes were depleted from the metastatic fluids by using density gradient Lymphoprep (Stemcell Technologies) following manufacturer's protocol. Clinical information about patient samples is shown in **Table S4**.

Breast cancer cell lines

MCF-7 parental, Tamoxifen- and Fulvestrant-resistant cell lines were a kind gift from Dr Julia Gee (University of Cardiff, Wales) and were cultured as previously reported (Simões et al., 2015). Cell lines were grown in monolayer in the presence of 10 ng/ml recombinant human IL1β (201-LB, R&D systems) and treated with 10 µg/ml Anakinra (Amgen, Cambridge, UK) or vehicle for 72 Hours prior to plating in mammosphere culture.

ALDH+/- cell isolation

Breast cancer cells were re-suspended in Aldefluor buffer and incubated in the presence of the Aldefluor reagent bodipyaminoacetaldehyde (BAAA) (Aldefluor assay, Stemcell Technologies) for 40 minutes at 37ºC, following the manufacturer's protocol. A subset of cells was also incubated with the selective ALDH inhibitor diethylaminobenzaldehyde (DEAB) to distinguish between

ALDH+ and ALDH- cells. When performing single-cell experiments using the C1 system (Fluidigm), cells were stained for CD44 (CD44-APC; BD,) and CD24 (CD24-PECY7) expression as well as ALDH activity in order to isolate ALDH+ cells that are not CD44^{high} CD24^{low}. Following incubation, cells were washed with PBS and stained with the cell viability dye 7-aminoactinomycin (7AAD, BD). Cells were then FACS-sorted into 200 μl of 2% Fetal Bovine Serum in Hank's Balanced Salt Solution (HBSS) using the InFlux (BD bioscience). Single colour stains were included for compensation and gating purposes. Data was analysed using FlowJo 10.1.

Mammosphere culture

Cells from primary samples were seeded at a density of 500 cells/cm^2 in 6-well polyHEMA (Poly (2-hydroxyethylmethacrylate)) coated plates containing mammosphere media (DMEM/F12 media with L-Glutamine (Gibco), B27 supplement (Gibco; 12587) and 20 ng/ml EGF (Sigma)). Similarly, MCF-7 cells were seeded at a density of 200 cells/cm^2. Cells were cultured for 7 days (primary samples) or 5 days (cell lines) at 37ºC before counting mammospheres greater than 50 μm. Mammosphere forming efficiency (MFE) was calculated by dividing the number of mammospheres by the number of cells seeded per well and is expressed as the mean percentage of MFE (Shaw et al., 2012). For patient samples, experiments were carried out with at least 3 technical replicates (where possible, depending on the number of cells available after FACS-sorting). For cell line experiments, each experiment represents at least 3 technical replicates and three biological repeats.

Transplantation assays

In vivo studies were carried out in accordance with the UK Home Office (Scientific Procedures) Act 1986 under project licence PPL40/3645 and study protocols were approved by the CRUK Manchester Institute Animal Welfare and Ethical Research Board (AWERB).

MCF-7 cells were treated *in vitro* with 10^{-6} M 4-Hydroxytamoxifen (Sigma-Aldrich, H7904), 10^{-7} M fulvestrant (ICI 182,780, Tocris, 1047) or ethanol (vehicle) for 6 days following staining with the Aldefluor assay. Serial limiting dilution of sorted ALDH+ and ALDH- cells (10,000; 1,000; 100 cells) were resuspended in mammosphere media mixed 1:1 with Matrigel (BD bioscience, 356234) and inoculated subcutaneously into the left and right flanks of female NOD/SCID Gamma (NSG) mice. All in vivo work was carried out using n=4 mice for each condition. 90-day slow release estrogen pellets were implanted subcutaneously into mice before cell injection (0.72 mg, Innovative Research of America) and, after day 90, 8 μg/ml of 17-beta estradiol was administered in drinking water. Tumour measurements were taken three times a week and tumour size was calculated using the formula:

$$\text{Tumour size} = 0.5 \times \text{Length} \times \text{Width}^2$$

Positive tumour growth was assessed at week 20 after cell injection by determining the mice bearing a tumour greater than 300 mm^3. Extreme Limiting Dilution Analysis (ELDA) was performed using software available at http://bioinf.wehi.edu.au/software/elda/ (The Walter and Eliza Hall Institute of Medical Research) to assess differences in stem cell frequency.

RNA extraction and Real-Time PCR

ALDH+ bulk cells (≥10,000 cells) were sorted into 100 μl of lysis buffer containing 1% β-Mercaptoethanol, following by cell disruption and homogenisation via vortexing for 1 minute. RNA was extracted using the RNeasy Plus Micro Kit (Qiagen, 74034) with on-column DNase treatment following manufacturer's protocol. The Bioanalyzer (Agilent 2100 Bioanalyzer system, Agilent Technologies) and the Qubit (Thermofisher Scientific) were used for quantitation and quality control of the RNA.

Bulk transcriptome analysis

Human Array Gene 1.0 ST (Affymetrix) GeneChips were used to assess mRNA expression profile in bulk ALDH+ and ALDH- cells. Double stranded amplified cDNA was generated using the Ovation Pico WTA System V2 (NuGen) and the Single Primer Isothermal Amplification (SPIA) following manufacturer's guidelines. cDNA was fragmented and labelled prior hybridisation onto the array (GeneChip hybridization Oven 640, Affymetrix). The GeneChip array was then washed and stained using the Fluidics Station protocol FS450_0007 and the Affymetrix GeneChip Command Console Software (Affymetrix) following manufacturer's guidelines. The GeneChip array was scanned using the Scanner 3000 system with autoloader (Affymetrix).

Microarray data from cell line and patient samples were processed using the *Affy* package in R (Gautier et al., 2004). Data was quantile-normalised and Log2 transformed. Differential gene expression analysis was carried using paired Rank Products (Breitling et al., 2004). Meta-analysis was performed using iPathwayGuide (AdvaitaBio). Statistical significance for RNA expression was assessed using t-test parametric testing.

Single-cell data analysis

The following 68 genes were used for single-cell targeted transcriptome analysis.

ABCG2	*CDH3*	*FBXW7*	*IGFBP5*	*LIN28A*	*NOTCH3*	*TAZ*
AKT1	*CTNNB1*	*GAPDH*	*IL1R1*	*MET*	*NUMB*	*TGFB1*
ALDH1A3	*CXCR1*	*GATA3*	*IL6R*	*MKI67*	*PCNA*	*TGFBR1*
AR	*CXCR4*	*GJA1*	*IL6ST*	*MKP1*	*PGR*	*TP53*
BRCA1	*CYR61*	*GPRC5A*	*ITGA6*	*MTOR*	*PIK3CA*	*TSPAN6*
CA12	*DLL1*	*GSK3B*	*JAG1*	*MUC1*	*POU5F1*	*TWIST1*
CCND1	*ENAH*	*HER2*	*JAG2*	*NANOG*	*RAB7A*	*UXT*
CD24	*EPCAM*	*HES1*	*KRT18*	*NFKB1*	*SNAI2*	*YAP1*
CD44	*ESR1*	*HPRT1*	*KRT19*	*NOTCH1*	*SOCS3*	
CDH1	*EZH2*	*ID1*	*KRT8*	*NOTCH2*	*SOX2*	

Data generated by the Biomark (Fluidigm) were converted into Log2 expression values and quality controls were undertaken. These included data filtering to remove all values under the limit of detection, which was set to threshold cycles (Ct) greater than 28; the removal of genes expressed in 3 or less cells within each treatment and, the removal of outliers (via the function identifyOutliers implemented in the R package FluidigmSC - Fluidigm Corporation, 2014). Missing completely at random values were estimated and inputted using the R package MICE (Azur et al., 2011). Principal Component Analysis revealed a batch effect between experiments that was corrected using ComBAT implemented within the sva package in R (Leek et al., 2019). We undertook a statistical approach to eliminate doublets derived from equipment unfitness (Fluidigm Corporation, 2016). Using the package Mclust in R we fitted Gaussian mixture models to identify cell clusters within each treatment. These models indicated the existence of two very well defined cell clusters in each condition. The nature of these clusters was further investigated by plotting the average Log2 expression per gene in both clusters, pointing towards a stratification into

doublets and singlets. Clusters corresponding to singlets were taken forward for the analysis. This corresponds to 444 cell signals from 377 cells, proportions shown in table below. 96% of replicate analyses assigned cells to the same population (population A, population B or fulvestrant 7).

Experiment	Untreated cells	Tamoxifen treated cells	Fulvestrant treated cells
1	35	9	49
2	96	28	80
3	34	11	35
3 – Technical Replicates	31	9	27

With the aim of identifying different cell populations within treatment we used a finite Gaussian mixture model to (a) estimate the number of clusters within the data (function Mclust within the R package Mclust (Scrucca et al., 2016)) and (b) generate those clusters. Ward hierarchical clustering with bootstrapping (Ward, 1963) was undertaken with the package pvclust in R to find similarities between identified clusters and merge smaller clusters into larger ones (threshold of Approximately Unbiased (AU) p-value greater than 0.9). Merged clusters were assessed for biases regarding batch and plate and it was confirmed that they were not linked due to experimental handling. Further analysis of cluster similarities and genes associated to cluster differences were undertaken using Discriminant Analysis of Principal Components (DAPC) (Jombart et al., 2010) of the 7 clusters identified with Mclust and fitting a model built using 40 principal components (PCs) and 8 linear discriminants. The number of PCs to use to build the model was determined via cross-validation by building 1000 different models per PC with an 80-20 split of training/test data and selecting the combination that provided the maximum correct predictions with the lowest number of PCs. Further merging of the clusters was further

assessed using DAPC to find the differences between the three main populations of cells identified (A, B and Fulv7) and built with 40 PCs and 6 linear discriminants. Most important genes to discriminate the cell populations were determined using non-parametric Mann-Whitney test. False discovery rate was controlled via Benjamini and Hochberg method.

shRNA knockdown

The inducible Dharmacon TRIPZ lentiviral shRNA was used to stably down-regulate ALDH1A3 mRNA expression levels (ALDH1A3KD - Dharmacon, V3THS_378581; V3THS_378584; V3THS_378585).

Statistical analysis

P values less that 0.05 were considered significant (*p<0.05, **p<0.01, ***p<0.001). Results are presented as the mean of at least 3 independent experiments ± Standard Error of the Mean (SEM) or Standard Deviation (SD).

SUPPLEMENTAL REFERENCES

Azur, M. J., Stuart, E. A., Frangakis, C., Leaf, P. J. (2011). Multiple Imputation by Chained Equations: What is it and how does it work? Int J Methods Psychiatr Res 20, 40-49.

Breitling, R., Armengaud, P., Amtmann, A., Herzyk, P. (2004). Rank products: a simple, yet powerful, new method to detect differentially regulated genes in replicated microarray experiments. FEBS Lett 573, 83-92.

Fluidigm Corporation (2014). Fluidigm® SINGuLAR™ Analysis Toolset 2.0 R package. v3.6.2

Fluidigm Corporation (2016). Doublet Rate and Detection on the C1 IFCs White Paper, PN 101-2711 A1.

Gautier, L., Cope, L., Bolstad, B. M., Irizarry, R. A. (2004). affy--analysis of Affymetrix GeneChip data at the probe level. Bioinformatics 20, 307-315.

Jombart, T., Devillard, S., Balloux, F. (2010). Discriminant analysis of principal components: a new method for the analysis of genetically structured populations. BMC Genet 11, 94.

Leek, J. T., Johnson, W. E., Parker, H. S., Fertig, E. J., Jaffe, A. E., Storey, J. D., Zhang, Y., Torres, L. C. (2019). sva: Surrogate Variable Analysis. R package version 3.34.0.

Shaw, F. L., Harrison, H., Spence, K., Ablett, M. P., Simões, B. M., Farnie, G., Clarke, R. B. (2012). A detailed mammosphere assay protocol for the quantification of breast stem cell activity. J Mammary Gland Biol Neoplasia 17, 111-117.

Scrucca, L., Fop, M., Murphy, T. B., Raftery, A. E. (2016). mclust 5: Clustering, Classification and Density Estimation Using Gaussian Finite Mixture Models. R J 8, 289-317.

Ward, J. H. (1963). Hierarchical Grouping to Optimize an Objective Function. Journal of the American Statistical Association 58, 236-244.
